# An Integrative Model of Ion Regulation in Yeast

**DOI:** 10.1371/journal.pcbi.1002879

**Published:** 2013-01-17

**Authors:** Ruian Ke, Piers J. Ingram, Ken Haynes

**Affiliations:** 1 Department of Mathematics, Imperial College London, London, United Kingdom; 2 Centre for Integrative Systems Biology (CISBIC), Imperial College London, London, United Kingdom; 3 Department of Microbiology, Imperial College London, London, United Kingdom; Institute for Systems Biology, United States of America

## Abstract

Yeast cells are able to tolerate and adapt to a variety of environmental stresses. An essential aspect of stress adaptation is the regulation of monovalent ion concentrations. Ion regulation determines many fundamental physiological parameters, such as cell volume, membrane potential, and intracellular pH. It is achieved through the concerted activities of multiple cellular components, including ion transporters and signaling molecules, on both short and long time scales. Although each component has been studied in detail previously, it remains unclear how the physiological parameters are maintained and regulated by the concerted action of all components under a diverse range of stress conditions. In this study, we have constructed an integrated mathematical model of ion regulation in *Saccharomyces cerevisiae* to understand this coordinated adaptation process. Using this model, we first predict that the interaction between phosphorylated Hog1p and Tok1p at the plasma membrane inhibits Tok1p activity and consequently reduces Na^+^ influx under NaCl stress. We further characterize the impacts of NaCl, sorbitol, KCl and alkaline pH stresses on the cellular physiology and the differences between the cellular responses to these stresses. We predict that the calcineurin pathway is essential for maintaining a non-toxic level of intracellular Na^+^ in the long-term adaptation to NaCl stress, but that its activation is not required for maintaining a low level of Na^+^ under other stresses investigated. We provide evidence that, in addition to extrusion of toxic ions, Ena1p plays an important role, in some cases alongside Nha1p, in re-establishing membrane potential after stress perturbation. To conclude, this model serves as a powerful tool for both understanding the complex system-level properties of the highly coordinated adaptation process and generating further hypotheses for experimental investigation.

## Author Summary

Ion regulation is fundamental to cell physiology. The concentrations of monovalent ions, such as H^+^, K^+^ and Na^+^, determine many physiological parameters such as cell volume, plasma membrane potential and intracellular pH. In yeast cells, these parameters are maintained within a narrow range during the adaptation to external perturbations, including ionic, osmotic and alkaline pH stress. This is achieved by the remarkably coordinated activities of ion transporters, regulatory molecules and signaling pathways. The response characteristics of individual components in adaptation have been studied extensively. However, a coherent understanding of the coordinated adaptation process is lacking. In this study, we address this gap by constructing a mathematical model that integrates the characteristics of the ion transporters, regulatory molecules, signaling pathways and changes in cell volume. Using this model, we characterize the impact of ionic, osmotic and alkaline pH stresses on cellular physiology and analyze the role that individual components play in the cellular adaptation processes. Our results also reveal system level properties achieved by the concerted regulatory responses. Therefore, this integrated model serves as a suitable tool to understand the coordinated processes of ion regulation in response to environmental stresses, and to make predictions that are experimentally testable.

## Introduction

A feature of fungal physiology is the ability to adapt successfully to a variety of environmental perturbations, including ionic [1], osmotic [2] and pH stress [3]. All of these stresses have a substantial impact on intracellular K^+^/Na^+^ concentrations and other important physiological parameters of the cell such as cell volume, plasma membrane potential and intracellular pH [4]. These physiological parameters are fundamental for the proper function of cellular processes, such as protein synthesis and cell cycle progression, and often, they are interlinked with each other [5]. For example, cell volume determines the concentration of molecules, including K^+^, Na^+^ and H^+^, and the levels of intracellular and extracellular K^+^/Na^+^ have large impacts on intracellular pH, plasma membrane potential and enzyme activities [6]. Therefore, one of the key aspects of cellular adaptation to environmental stresses is the maintenance of these parameters within a narrow range. This is achieved through the orchestrated activity of monovalent ion transporters, regulatory enzymes, signaling pathways and cellular osmolyte metabolism (see Ref. 5 for a comprehensive review).

**Figure 1:**
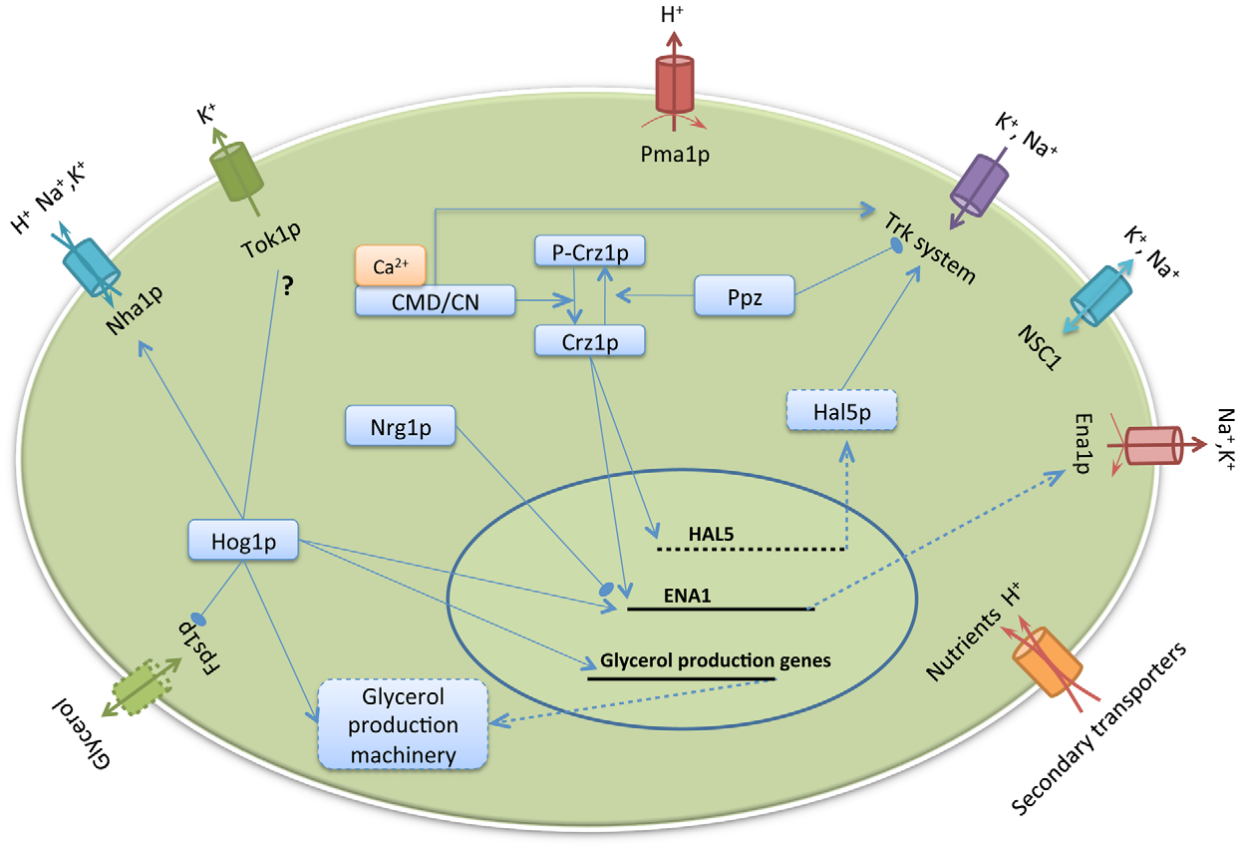
A schematic overview of the integrated model of *S. cerevisiae* ion regulation. The transporters modeled explicitly are Pma1p, Ena1p, Nha1p, Tok1p, the Trk system and the as yet genetically uncharacterized non-selective cation channel NSC1. The regulatory components explicitly considered for these transporters are Hog1p, calcineurin (CN), Ppz proteins and Nrg1p. Osmotic stress activates the protein kinase Hog1p, which regulates a variety of cellular components. All the four stress conditions considered in this study, i.e. NaCl, osmotic, KCl and alkaline pH stresses, lead to increase in the level of cytosolic Ca^2+^, which activates calcineurin. Under alkaline pH stress, the production of Nrg1p is inhibited. *ENA1* expression is upregulated under stress conditions through activations of Hog1p and calcineurin or inhibition of Nrg1p. H^+^ uptake through secondary transporters is modeled as a measure of the level of nutrient intake in a cell. See Table 1 for descriptions of the interactions considered in this study. Components that are not modeled explicitly are denoted as dashed rectangles. Lines with arrows and stops connecting components denote activation and inhibition, respectively. Solid and dashed lines denote regulation and gene expression, respectively. The question mark between the interaction of Hog1p and Tok1p denotes that the effect of this interaction on Tok1p is unknown.

The major monovalent ion transporters at the plasma membrane in budding yeast, *Saccharomyces cerevisiae*, include Pma1p, Tok1p, Nha1p, Ena1p and the Trk1,2p (Trk) transporter system [5] (Fig. 1). Pma1p is an H^+^-ATPase, which extrudes intracellular H^+^ produced by cellular metabolism [7]. This extrusion creates a proton gradient across the plasma membrane, which serves as an energy source for molecular transport. K^+^ is essential for maintaining normal cellular functions. It is taken up primarily through the Trk transporter system [8]. Due to its similarity with K^+^ and its abundance in natural environment, Na^+^ enters the cell through different K^+^ transporters. High intracellular Na^+^ is toxic to the cell. To extrude excessive Na^+^ and sometimes K^+^, the yeast cell uses three different transporters: Nha1p, Ena1p and Tok1p. Nha1p is a Na^+^,K^+^/H^+^ antiporter that extrudes Na^+^ and K^+^ by taking in H^+^ across the plasma membrane [9]. Ena1p is a Na^+^/K^+^-ATPase that extrudes Na^+^ and K^+^ using the energy released through ATP hydrolysis [10]. Tok1p is a voltage-gate channel that extrudes K^+^ exclusively [11]. Under ionic stress conditions, cellular adaptation has been shown to involve both immediate and long-term regulation of these monovalent cation transporters [6], [12], [13]. This includes post-translational regulation of Nha1p, Tok1p and the Trk system and transcriptional regulation of Ena1p through the activations of the HOG pathway and the calcineurin pathway.

Because of the importance of these transporters, and the regulatory mechanisms that control their function, in maintenance of cellular homeostasis, they have been subjected to intensive study. Substantial progress has been made in characterizing the response of individual transporters [14], [15] and individual regulatory signaling events [16], [17]. These studies revealed that each transporter has distinct response characteristics and is regulated by different pathways. However, the exact role that each transporter plays in maintaining cellular physiological parameters, such as intracellular K^+^/Na^+^ concentration, intracellular pH and the plasma membrane potential, is still not well understood. This is partly because a large number of transporters are involved in transporting relative few types of ions (K^+^, Na^+^ and H^+^), and consequently, the impact of one transporter is dependent on the activity of other related transporters. Therefore, investigations focusing on a single transporter or pathway may not be sufficient to reveal how transporters and regulatory mechanisms collectively contribute to the maintenance of the physiological parameters, and how they are coordinated to ensure a robust response to stress conditions. Therefore, a systems-level approach that integrates available data on individual components to understand the system-level properties of ion regulation process is needed [5].

To attempt to address these questions, we have constructed an integrative model of ion regulation in *S. cerevisiae*. Mathematical modeling has proven to be a promising tool for the study of the complex processes of environmental stress adaptation [16], [17] and especially, molecular transporter and ion regulation [18], [19]. This approach has been fruitful in revealing how the system level properties emerged from collected activities of individual components and identifying the role each component plays in the system [18], [19], [20], [21]. This approach seems particularly well suited to understanding the coordinated activities of various components, in this case ion transporters and their regulatory mechanisms, and allows the impact of other factors such as cell volume, intracellular ion concentration and membrane potential to be included in analyses. Using the integrative model, we specifically investigate: 1) the different roles of the three K^+^/Na^+^ exporters, i.e. Tok1p, Nha1p and Ena1p, in maintaining cation homeostasis; 2) the role of transcriptional regulation, specifically via activation of the HOG and calcineurin pathways; 3) how they are interconnected to maintain fundamental physiological parameters such as membrane potential and intracellular pH and 4) the overall strategies employed by the cell to ensure a robust response to four stress conditions that are most relevant to ion homeostasis and have been under extensive study previously, i.e. NaCl, osmotic, KCl and alkaline pH stresses.

## Results

### Model overview

The integrative mathematical model describes *S. cerevisiae* cellular physiological parameters, including intracellular cation concentrations (H^+^, Na^+^ and K^+^); plasma membrane potential; cell volume and regulatory responses (post-translational and transcriptional) to external osmotic, ionic and alkaline pH perturbations (Fig. 1). This integrated model can be categorized into three linked modules: the ‘transporter’ module, the ‘signaling’ module and the ‘volume’ module. The ‘transporter’ module incorporates the dynamics of intracellular cation concentration, the characteristics of cation flux through transporters at the plasma membrane and the activities of enzymes regulating these transporters. Six transporters were considered explicitly in this module: Pma1p, Tok1p, Nha1p, Ena1p, the Trk1,2p (Trk) transporter system, and the as yet genetically uncharacterized non-selective cation channel NSC1 (Table 1). In addition, H^+^ uptake through other secondary transporters was also considered. These secondary transporters take up external nutrient molecules into the cell using electro-chemical energy stored in the proton gradient. The ‘signaling’ module keeps track of the activities of stress response signaling pathways (the HOG pathway and the calcineurin pathway), regulatory enzymes (Nrg1p), the expression levels of *ENA1* and the changes in the intracellular concentration of the osmostablizer, glycerol. The ‘volume’ module describes the volume changes of the cell depending on internal and external osmotic pressures.

**Table 1:** Descriptions of the cellular molecules considered in the integrated model.

Name	Description	Functions considered in this study	Ref.
**Pma1p**	H^+^-ATPase at the plasma membrane	H^+^ extrusion	[7]
**Trk system**	Trk1,2p; major K^+^ uptake transporters	Medium affinity and high affinity K^+^ uptake	[8], [52]
**Tok1p**	Outward-rectifier K^+^ channel of the plasma membrane	K^+^ extrusion	[11]
**Nha1p**	Na^+^/H^+^ antiporter involved in Na^+^ and K^+^ efflux through the plasma membrane	Na^+^ and K^+^ extrusion and H^+^ uptake	[9]
**Ena1p**	P-type Na^+^-ATPase	Na^+^ and K^+^ extrusion	[10], [25]
**NSC1**	non-specific cation channel	Na^+^ and K^+^ channel	[53]
**Nutrient transporters**	Secondary transporters responsible for nutrient uptake	H^+^ uptake	[35], [36]
**Ppz**	Ppz1,2p; Serine/threonine protein phosphatases	Sensitive to intracellular pH; Deactivation of Crz1p; switch Trk system from a high affinity state to a medium affinity state	[6], [51], [54], [55]
**Hog1p**	Mitogen-activated protein kinase involved in osmoregulation	Interaction with Nha1p and Tok1p at the plasma membrane; regulation of cell volume through interaction with glycerol production machineries and genes.	[13], [17], [56]
**Glycerol production genes**	Metabolic genes involved in glycerol production.	Glycerol production	[17], [23], [57]
**Calmodulin/Calcineurin (CMD/CN)**	a calcium-binding messenger protein/a calcium-dependent serine-threonine phosphatase	They are activated by elevated level of cytosolic Ca^2+^ under NaCl, osmotic, KCl and pH stress conditions. They activate Crz1p; switches Trk system from a medium affinity state to a high affinity state; increases Trk activity. Addition of FK506 blocks the activity of calcineurin.	[31], [50], [57], [58]
**Crz1p**	Transcription factor regulated by calcineurin	Up-regulation of ENA1 expression	[59], [60]
**Nrg1p**	Stress responsive transcription factor	Inhibition of ENA1 expression	[40]
**ENA1**	Gene encoding Ena1p	Under the regulation of the HOG pathway, the calcineurin pathway (Crz1p) and Nrg1p	[12]

### Model construction and validation

An inherent challenge with large models that integrate several cellular processes is that it is usually very difficult to estimate parameter values simultaneously from a limited number of data sets in a statistically meaningful way. However, by ensuring consistency with experimental data at each step of model construction, several integrative models constructed previously have shown notable successes in predicting biological mechanisms (for example, see refs. [17], [22]). Here, we have adopted a similar approach (Fig. S1). In this section we describe the model construction, integration and validation briefly. The detailed model for each module is described in the Methods section, Table S1, S2, S3, S4, S5 and Text S1.

The ‘transporter’ module was composed of sub-modules that describe each ion transporter involved in monovalent ion homeostasis, whereas the ‘signaling’ module was composed of sub-modules that describe the signaling pathways that regulate those transporters. We first constructed models for each of the sub-module such that they are consistent with published experimental data or adapted from previously published models with minimal modification. For example, the sub-modules for the transporter Tok1p, the Hog1p pathway and cell volume were adapted from works by Loukin and Saimi, Klipp *et al.* and Zi *et al.* [15], [17], [23].

**Figure 2:**
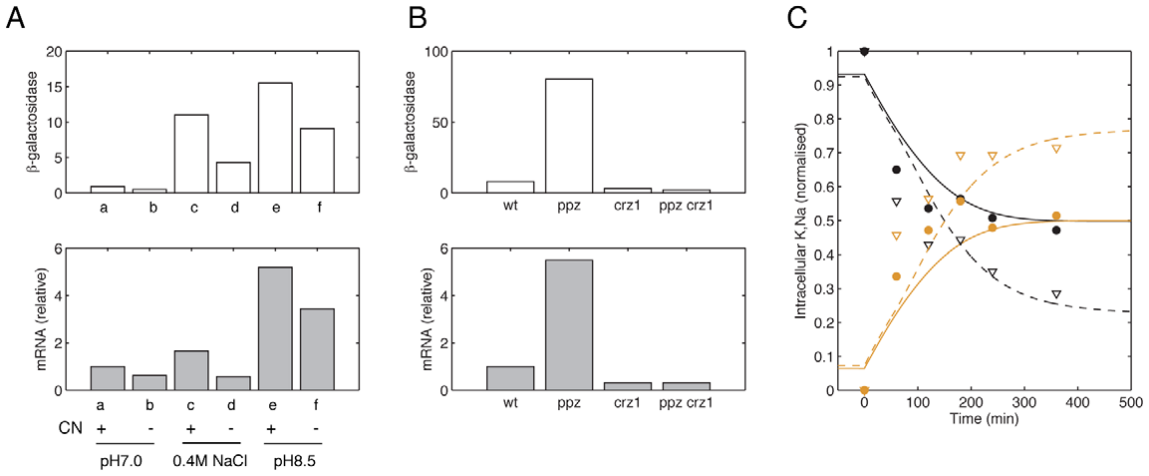
Published data sets used in integration of the model and corresponding simulation results. (**A**) Experimentally measured *ENA1* transcription activities [50], (upper panel) and simulated mRNA levels (lower panel) in wild type cells (a,c,e) and *cnb1* mutant (b,d,f) under different stress conditions: pH 7.0 (a,b), 0.4M NaCl (c,d) and pH 8.5 (e,f). Simulation results were obtained 30 minutes after stress in accordance with the experiments performed in ref. 46, and were normalized to the value for the wild type cell at pH 7.0 (a). (**B**) Experimentally measured *ENA1* transcription activities (Ref. [51], upper panel) and simulated steady state levels of *ENA1* mRNA (lower panel) in wild type cells, *ppz1*, *crz1* and *ppz1crz1* mutant cells. Simulation results are normalized to the value for the wild type cell. (**C**) Experimentally measured [26] and model simulated time courses of the amount of intracellular K^+^ and Na^+^ in wild type cells and cells treated with calcineurin inhibitor FK506. Experimental data and simulation results are normalized to their total cation (K^+^ and Na^+^) concentrations at time point 0 minute, respectively. Circles and triangles denote data measurements: K^+^ (black circle) and Na^+^ (orange circle) in wild type cells, K^+^ (black triangle) and Na^+^ (orange triangle) in cells treated with FK506. Simulation results for K^+^ (black lines) and Na^+^ (orange lines) are shown in solid lines for wild type cells and dashed lines for cells treated with FK506.

The models for each sub-module were determined based on data measuring the activity of individual components. To integrate these sub-modules and ensure that they are linked coherently in the integrated model, we surveyed the literature and chose three data sets to further constrain the parameters in the integrated model (Fig. 2). A subset of parameter values that determine the total activity of each sub-module was then adjusted such that the simulation results of the integrative model are consistent with these three data sets. Specifically, these parameters were the rate constants determining the total flux through each transporter and those parameters that determine the extent of *ENA1* expression in response to activations of different signaling pathways (see Table S2 and S3). This subset of parameters was chosen such that the relative contribution of each sub-module to overall ion homeostasis was adjusted while the response characteristics of each sub-module were kept the same.

By adjusting the model based on the three data sets, we show that the integrated model is in a good agreement with the experimental measurements (Fig. 2). The first two data sets compare the contribution of different signaling pathways to the regulation of *ENA1* gene expression in different genetic backgrounds i.e. wild-type cells, calcineurin knock-out (Fig. 2A) and ppz (ppz1,2) mutants (Fig. 2B). By comparing model simulations to these two experiments, we ensure that the relative contributions of the Ppz phosphatases, the HOG pathway and the calcineurin pathway (under NaCl stress) and Nrg1p (under alkaline pH stress) to the transcriptional up-regulation of *ENA1* are correctly integrated in the model. The third data set measures the dynamics of intracellular K^+^ and Na^+^ concentrations under NaCl stress, in the presence or absence of the calcineurin inhibitor, FK506 (Fig. 2C). By comparing the model with this data set, we ensure that the models describing the activities of those transporters involved in the K^+^/Na^+^ regulation, i.e. the Trk system, Nha1p, Ena1p, Tok1p and NSC1, are correctly integrated in the model.

**Figure 3:**
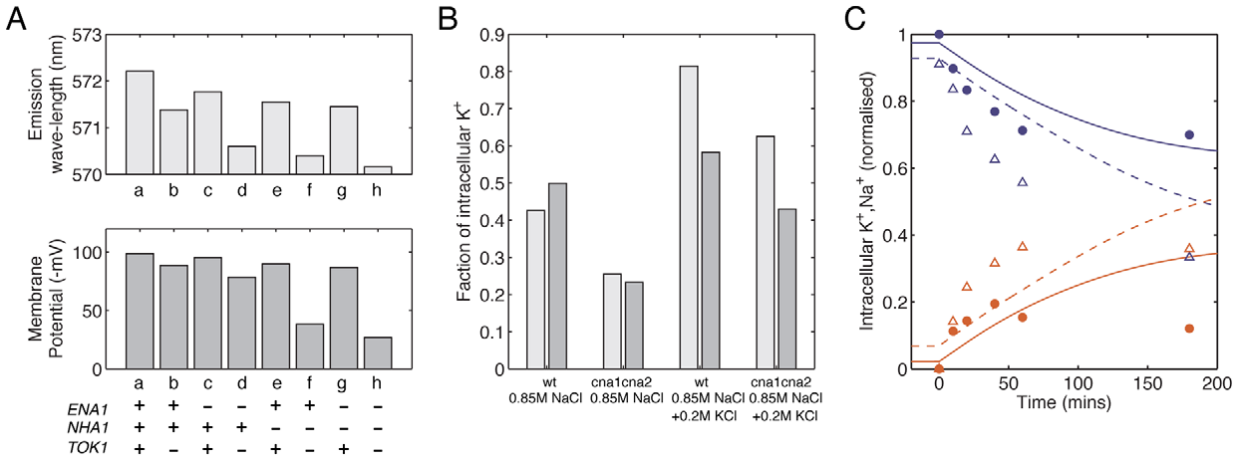
The model correctly predicts biology. Three published data sets, not used for model building were used to validate the model. (**A**) Experimentally measured emission wavelengths [24] (top panel) reflecting membrane potentials and corresponding simulation results (bottom panel) for wild type and mutant cells. Strains as indicated in the figure. (**B**) Experimentally measured [26] and simulated Intracellular K+ concentrations for wild type and *cna1cna2* cells during 0.85M NaCl stress with or without 0.2M KCl. Data (light gray) and simulation results (dark gray) are normalized to their maximal values, respectively. (**C**) Experimentally measured [27] and model simulated time courses of the total amount of intracellular K+ (blue) and Na+ (red) for *HAL3* over-expression and *hal3* mutant strains during 0.85M NaCl stress. Previously published data are shown as closed circle for the *HAL3* over-expression mutant and as triangles for the *hal3* mutant. Simulation results are shown as solid lines for the *HAL3* over-expression mutant and as dashed lines for the *hal3* mutant. YPD were used to grow yeast cells in the three sets of experiments. In the model, external baseline Na^+^ and K^+^ concentrations are 0.1 mM, 5 mM, respectively, and the baseline external pH is 6.5.

To validate and test the predictive power of the integrated model, we compared the simulation results with the other three data sets that had not been considered during the model building process. The comparison allows us to test whether the relative contributions of those transporters are correctly integrated in the model (Fig. 3A) and whether the dynamics of calcineurin pathway and the transporters responsible for K^+^/Na^+^ homeostasis is well approximated under stress conditions in both wild-type and mutant cells (Fig. 3B, C). In the first experiment (Fig. 3A), the membrane potentials were measured in wild-type cells and mutant cells where *ENA1-4*, *NHA1*, *TOK1* and combinations of these genes were knocked out [24]. Simulation of the model for the wild-type and the mutant cells agreed well with experimental measurements (Fig. 3A), suggesting the relative activities of these three pumps are well approximated in the model (note that in the model we only consider the effect of Ena1p among proteins encoded by *ENA1-4* since Ena1p is the primary Na^+^-ATPase responsible for Na^+^ extrusion in budding yeast [25]). The second and the third experiments measure the impact of combinations of stress conditions for both wild-type and calcineurin mutant cells [26] (Fig. 3B) and the intracellular K^+^/Na^+^ concentrations in both *HAL3* over-expression and deletion mutants during NaCl stress [27] (Fig. 3C), respectively. The halotolerance protein Hal3p binds to Ppz phosphatases, and over-expression and deletion of *HAL3* in a cell decreases and increases the activity of Ppz proteins, respectively, which in turn affects a variety of cellular components including the Trk K^+^ uptake system and calcineurin activity [6]. The model simulation in general agreed with experimental data, confirming the accuracy of the model in predicting the regulatory activity of calcineurin and the K^+^/Na^+^ homeostasis.

To gain further insight into how well the parameters in our model are constrained, we evaluated the sensitivity of each sub-module model to the data shown in Fig. 2C, 3B and 3C where quantitative measurements are available (see Methods for detail). In general, the activities of three transporters, i.e. Ena1p, Tok1p and the Trk system, and the calcineurin pathway are tightly constrained by the experimental data shown in Fig. 2C as well as the data shown in Fig. 3B and 3C (Table S6). This suggests the integrative model serves as a good approximation to the dynamics of each cellular component. Note that although the activity of Pma1p, the dynamics of the HOG pathway and cell volume change are not constrained by these three data sets, we think our model describes those processes well. This is because the model for the Pma1p activity is calibrated with previous estimates and the model for the HOG pathway and the cell volume change was adopted from a previously established model which has been shown to be consistent with a wide range of experimental measurements [23] (see Text S1 for details).

Yeast cells are able to adapt to a remarkably wide range of stress conditions by employing both short-term and long-term regulatory responses. Among those stress conditions that yeast cells frequently encounter, the ones that are most relevant to ion regulation include NaCl, osmotic, KCl and alkaline pH stresses [5]. Therefore, in the following sections we use this model to investigate how different transporters, such as Ena1p, Tok1p and Nha1p, the HOG pathway and the calcineurin pathway are coordinated to achieve both immediate and longer-term adaptation to these stress conditions.

### Cytosolic activated Hog1p inhibits Tok1p activity to decrease membrane potential and intracellular Na^+^ as an immediate response to NaCl stress

We first used the model to determine the effect of Hog1p phosphorylation on Tok1p at the plasma membrane upon NaCl stress. Higher external Na^+^ imposes strong stresses on normal cellular processes. In particular, high intracellular Na^+^ concentration at the beginning of NaCl stress can cause transcription factors to dissociate from DNA. Proft and Struhl have suggested that the interaction of phosphorylated Hog1p (P-Hog1p) with the membrane localized Na^+^/H^+^ antiporter Nha1p and the potassium channel Tok1p decreases intracellular Na^+^, and thereby facilitates transcription factor rebinding to DNA in the first 10 to 30 minutes after initiation of stress [13]. It has been shown that phosphorylation of Nha1p by Hog1p increases the rate of Na^+^ efflux [13], [28]. However, it is unclear how the activity of Tok1p changes upon phosphorylation by P-Hog1p and how this change affects intracellular Na^+^.

**Figure 4:**
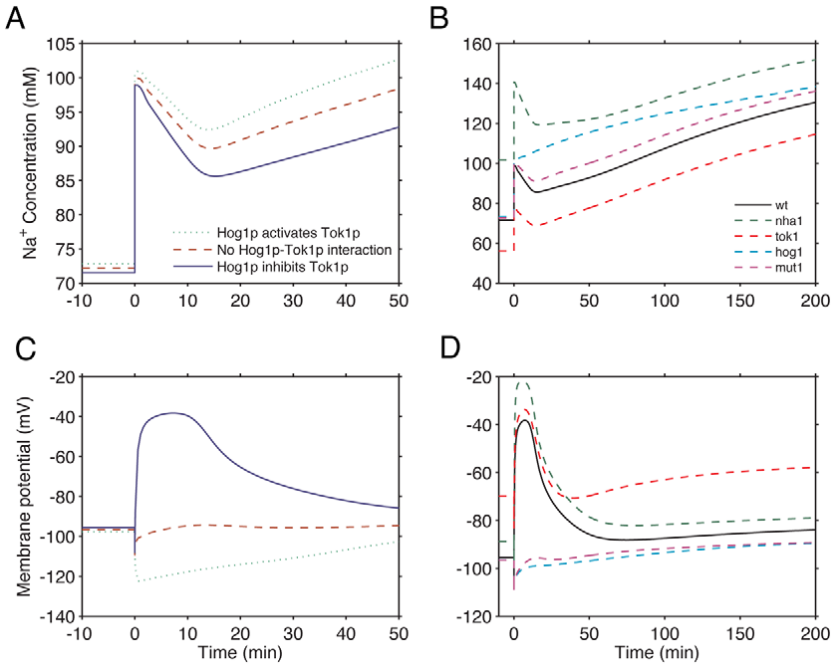
Phosphorylated Hog1p inhibits Tok1p activity and the interaction of phosphorylated Hog1p with Nha1p/Tok1p decreases intracellular Na^+^ and polarizes the plasma membrane. Time course simulations of (A & B) intracellular Na^+^ concentration and (C & D) plasma membrane potential respectively, under 0.4M NaCl stress for (**A**) three scenarios, 1) P-Hog1p activatesTok1p (dotted green line), 2) no Hog1p-Tok1p interaction (dashed red line) or 3) P-Hog1p inhibits Tok1p activity (solid blue line); (**B**) and in wild type (solid black) or *nha1* (dashed green), *tok1* (dashed red), *hog1* (dashed cyan) strains plus a hypothetical mutant where the interaction between P-Hog1 and Tok1p/Nha1p is abolished. (**C**) and (**D**) shows the plasma membrane potentials during the simulations in (A) and (B), respectively. Note that, in (B) and (D), cytosolic P-Hog1p inhibits Tok1p activity. External baseline Na^+^ and K^+^ concentrations are 5 mM, 1 mM, respectively, and the baseline external pH is 6.5.

To focus on the impact of the interaction of P-Hog1p with Tok1p on the initial adaptation process, we simulated the model with three scenarios for 50 minutes under 0.4M NaCl stress: (1) cytosolic P-Hog1p activates Tok1p; (2) no interaction occurs between P-Hog1p and Tok1p and (3) that cytosolic P-Hog1p inhibits Tok1p. Simulations for the scenario that Tok1p is inhibited by cytosolic P-Hog1p resulted in the lowest level of intracellular Na^+^ during the first 50 minutes of adaptation (Fig. 4A). The reason is that the membrane potential is highly dependent on the activity of Tok1p; inhibition of Tok1p activity resulted in a depolarized membrane, which in turn, reduces Na^+^ influx into the cell, whereas increasing Tok1p activity polarized the plasma membrane, thereby, resulting in around 10 mM higher intracellular Na^+^ concentration during the first 50 minutes (Fig. 4C). Therefore, by considering the collective activities of the monovalent transporters and the HOG pathway, our model predicts the inhibitory action of Hog1p on Tok1p. In the simulations below, we set this interaction as inhibition.

In the study of Proft and Struhl [13], the *nha1*, *tok1* and *hog1* deletion mutants showed different extents of delayed transcription factor rebinding to DNA under 0.4M NaCl stress. To further examine the impact of this interaction of P-Hog1p with Nha1p and Tok1p in the immediate adaptation to NaCl stress in a quantitative way and give insights into the experimental observation as to why transcription factor rebinding to DNA is delayed in these mutants, we simulated the model for the wild-type strain, the *nha1*, *tok1* and *hog1*deletion mutant strains plus a hypothetical strain (*mut1*) lacking interactions between P-Hog1p and Nha1p or Tok1p.

Higher intracellular Na^+^ concentrations were observed in simulations for the *nha1*, *hog1* and *mut1* mutants under 0.4MNaCl stress (Fig. 4B). More than 20 mM Na^+^ accumulated in *nha1* mutant cells even before NaCl stress onset, probably due to reduced Na^+^ efflux capacity. This difference resulted in a much higher intracellular Na^+^ concentration in *nha1* mutant cells (140 mM) than in wild-type cells (100 mM) at the beginning of the stress. The *hog1* mutant also showed a notably higher intracellular Na^+^ level throughout the simulation, due to the inability to restore cell volume. These results offered explanations as to why it takes longer for *hog1* and *nha1* strains to restore transcriptional activity at the beginning of NaCl stress [13]. There was also an approximately 10 mM higher Na^+^ concentration seen in the hypothetical strain where the interaction of Hog1p with Nha1p and Tok1p was abolished than in the wild-type strain during the first 120 minutes of stress, demonstrating the importance of this interaction. The *tok1* mutant had a lower intracellular Na^+^ level in the simulation due to the inability of the cell to establish the membrane potential under both unstressed growth conditions and under NaCl stress (Fig. 4B). This is not consistent with the observation of the delayed transcription factor rebinding [13]. We reason that disruption of Tok1p may induce defects that are associated with membrane depolarization or other aspects of cellular adaptation that are not investigated in this study.

The simulation predicts that intracellular Na^+^ concentration in wild type cells reached a higher level after 120 minutes, than seen at the onset of the stress. It has been shown that the vacuole plays an important role in NaCl stress adaptation and there are active Na^+^ transports from the cytosol to the vacuole under NaCl stress [29]. Therefore, it is likely that the Na^+^ concentration is kept at a low level in the cytosol and the nueclues under NaCl stress through sequestration of Na^+^ into the vacuole [30].

### The cellular responses to NaCl stress

Both NaCl and sorbitol are frequently used to investigate the osmotic stress response in experimental studies. However, NaCl imposes saline stress as well as osmotic stress. Here, we investigate the impact of NaCl stress on a cell in this section and the impact of osmotic stress in the next section. We focus on the roles of the Na^+^ exporters, i.e. Nha1p, Ena1p and the calcineurin pathway and how they are coordinated during the adaptation processes. In the simulations below, the external Na^+^ and K^+^ concentrations and the external pH are assumed to be 5 mM, 1 mM and pH 6.5, respectively, under unstressed condition. Changes in these external conditions do not alter the results qualitatively.

**Figure 5:**
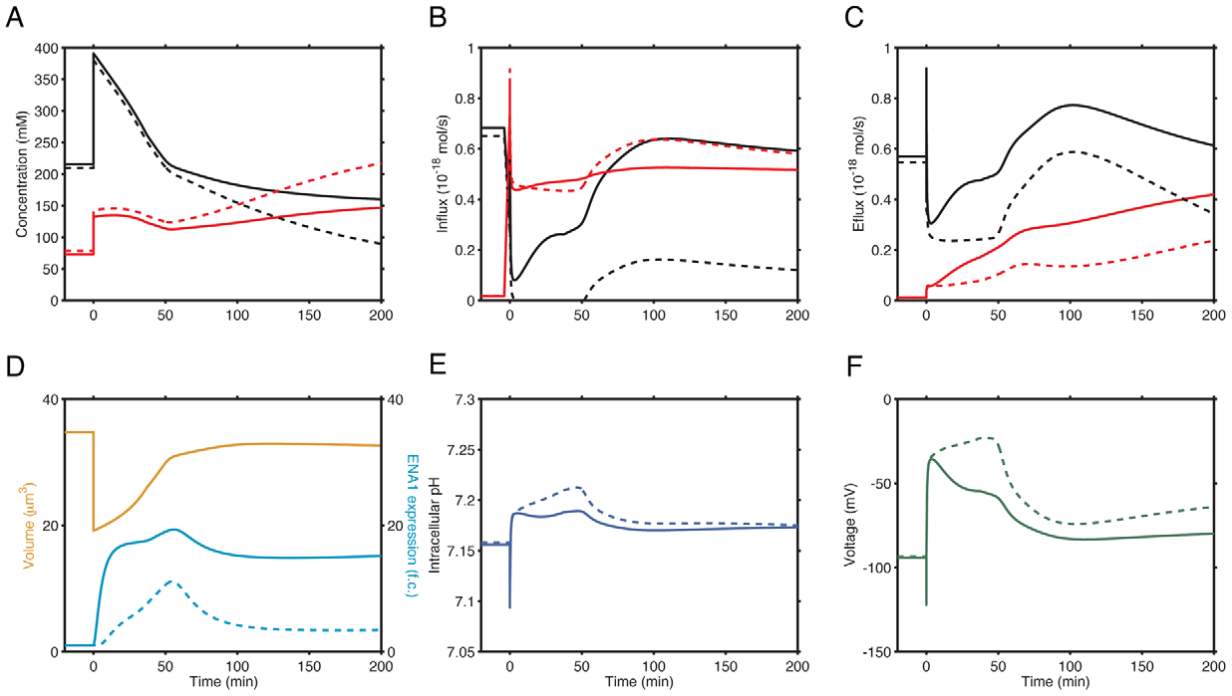
Cellular physiological parameters during time course simulation of 0.8M NaCl stress responses in wild type cells with or without the calcineurin inhibitor, FK506. (**A**) Intracellular K^+^ (black lines) and Na^+^ (red lines) concentrations in untreated (solid lines) and FK506 treated cells (dashed lines). (**B**) K^+^ (black lines) and Na^+^ (red lines) influx in untreated (solid lines) and FK506 treated cells (dashed lines). (**C**) K^+^ (black lines) and Na^+^ (red lines) efflux in untreated (solid lines) and FK506 treated cells (dashed lines). (**D**) Cell volume (orange lines) and *ENA1* expression (light blue lines) in wild type cells without (solid lines) and with FK506 (dashed lines). Addition of FK506 does not have any effect on cell volume. (**E**) Intracellular pH in wild type cells without (solid) and with FK506 (dashed). (**F**) Membrane potentials in wild type cells without (solid) and with FK506 (dashed). External baseline Na^+^ and K^+^ concentrations are 5 mM, 1 mM, respectively, and the baseline external pH is 6.5.

High osmolarity and high external Na^+^ concentration imposes substantial changes of several cellular physiological conditions. Simulation of the model shows that, upon NaCl stress, intracellular Na^+^ and K^+^ concentrations increased suddenly (Fig. 5A), concomitant with a decrease of cell volume (Fig. 5D). At the same time, due to high external Na^+^ concentration, Na^+^ influxes through Trk1p and NSC1 increased to a high level (Fig. S2).

As examined in the section above, the immediate responses to these changes are mediated, at least in part, by activation of Hog1p. Phosphorylation of Hog1p led to increased glycerol biosynthesis, which acts as an osmostabiliser and restores cell volume [16]. At the same time, the interaction between cytosolic P-Hog1p with Nha1p and Tok1p decreased intracellular Na^+^ concentration. Another consequence of inhibition of Tok1p was a depolarized membrane (Fig. 5E,F). This lowered membrane potential led to a reduced secondary transporter activity (as represented by H^+^ uptake shown in Fig. S2).

During the long-term cellular adaption to NaCl stress, the simulation showed a switch in transporter use for Na^+^ export at around 50 minutes after stress onset (Fig. S2). Upon initial imposition of NaCl stress, intracellular Na^+^ was extruded primarily by Nha1p, this rate decreased over time as a result of dephosphorylation of P-Hog1p, once cell volume was restored. Concurrently, the activity of Ena1p increased due to transcriptional up-regulation of *ENA1* expression (Fig. S2), maintaining Na^+^ extrusion. This is consistent with the major role of Ena1p during NaCl stress adaptation [12].

To examine the role of calcineurin activation, we simulated the model for knock-out mutant strains where calcineurin activity is abolished. This setting is also equivalent to cells treated with the immunosuppressant FK506, which inhibits calcineurin activity. We, therefore, refer to this set of simulations as simulations for cells treated with FK506 below. In the simulation, the expression of *ENA1* returned to the unstressed level after 100 minutes after stress initiation in cells treated with FK506. Consequently, Na^+^ concentrations approached 220 mM, in stark contrast to ∼150 mM Na^+^ in untreated cells (Fig. 5A). Notably, other consequences of the low *ENA1* expression in FK506 treated cells are a lower membrane potential, and consequently, a lower H^+^ uptake rate and a higher intracellular pH compared to wild-type cells (Fig. 5E,F and S2). Therefore, our simulation results give insight into the experimental observation that activation of calcineurin and consequently a high expression of *ENA1* is critical for the cell to maintain a relatively low intracellular Na^+^ concentration [26].

### The cellular responses to osmotic stress

To consider osmotic stress alone, we interrogated the model with an equivalent osmolarity environment lacking an ionic component, i.e. 1.6M sorbitol. This resulted in the same decrease in cell volume in the simulation, leading to a higher intracellular Na^+^ concentration (Fig. S3 and S4). The intermediate cellular response (in the first 0–30 minutes) to high osmolarity was very similar to the response to NaCl stress. However, Na^+^ influxes were at a low level in the long term in contrast to the high Na^+^ influx under NaCl stress (Fig. S3B). Consequently, Na^+^ concentration returned to unstressed levels once cellular volume was restored (Fig. S3A, D). High *ENA1* expression is not required for long term adaptation to osmotic stress, and FK506 had little effect on the adaptation response to osmotic stress, and in stark contrast to what is seen in cells treated with osmoequivalent levels of NaCl. Therefore, our model suggests that the role of calcineurin activation is to respond to the ionic stress induced by high external Na^+^ but not pure osmotic stress, consistent with previous studies [26], [31],

### Nha1p and Ena1p are potential regulatory targets to restore the membrane potential under KCl stress

Yeast cells grow well under KCl stress [9]. In our simulation, however, we found that 0.8M KCl stress led to a highly depolarized plasma membrane (the membrane potential was up to +20 mV) (Fig. S5), as a result of high K^+^ flux into the cell (Fig. S6). It is known that positive membrane potential is detrimental to the cell and induces severe nutrient limitation [4], [32]. We therefore reasoned that it is likely that unidentified regulatory mechanisms, not yet included in the model, are essential for restoring the membrane potential and thus adaptation to KCl.

**Figure 6:**
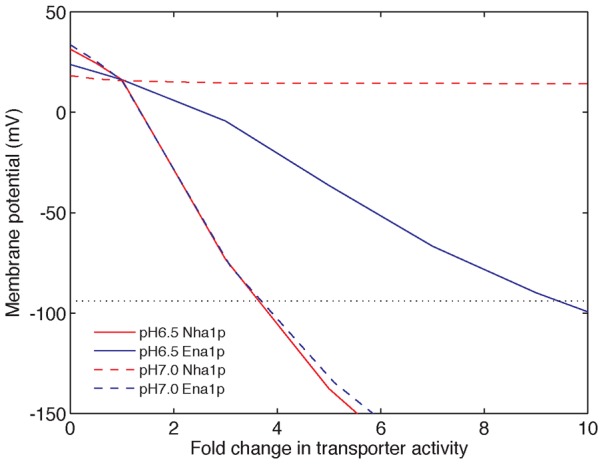
Increasing Nha1p and Ena1p activity restores the membrane potential under KCl stress at pH 6.5, whereas increasing Ena1p activity restores the membrane potential under KCl stress at pH 7.0. Red and blue solid lines denote simulations for increasing the activities of Nha1p and Ena1p under 0.8M KCl at pH 6.5, respectively. Red and blue dashed lines denote simulations for increasing the activities of Nha1p and Ena1p under 0.8M KCl at pH 7.0, respectively. The grey dashed line shows the membrane potential at the unstressed level. The values of the membrane potential and intracellular pH were taken at 200 minutes after stress.

To identify candidate regulatory targets, we systematically searched for transporters of which varying the activity resulted in increased protection against KCl stress. We found that, in slightly acidic environment (pH 6.5), increases in the activities of Nha1p and Ena1p had high impacts on the plasma membrane potential (Fig. 6). Nine-fold increase in Ena1p activity or four-fold increase in Nha1p activity restored the membrane potential to the unstressed level (around −94 mV). In contrast, in neutral environment (pH 7.0), only increases in the activity of Ena1p restored the plasma membrane potential, because the activity of Nha1p was much lower at pH 7.0 where the proton gradient is disrupted (Fig. 6). Interestingly, Banuelos *et al.* showed that, under KCl stress, Nha1p is required for growth at pH 6.5 and Ena1p is required for growth at pH 7.0, but currently the mechanism is unknown. [9]. Taken together, our results suggest that Nha1p and/or Ena1p are activated (through either post-translational or transcriptional regulation) under KCl stress conditions (although differently depending on external pH) by unidentified mechanisms, and we predict that this activation or upregulation is essential to restore the plasma membrane potential in the adaptation to KCl stress. Further experiments are needed to verify the predicted increases in Nha1p and Ena1p activities under KCl stress at different external pH and test the molecular mechanism that regulates these increases.

### The cellular response to alkaline pH stress

**Figure 7:**
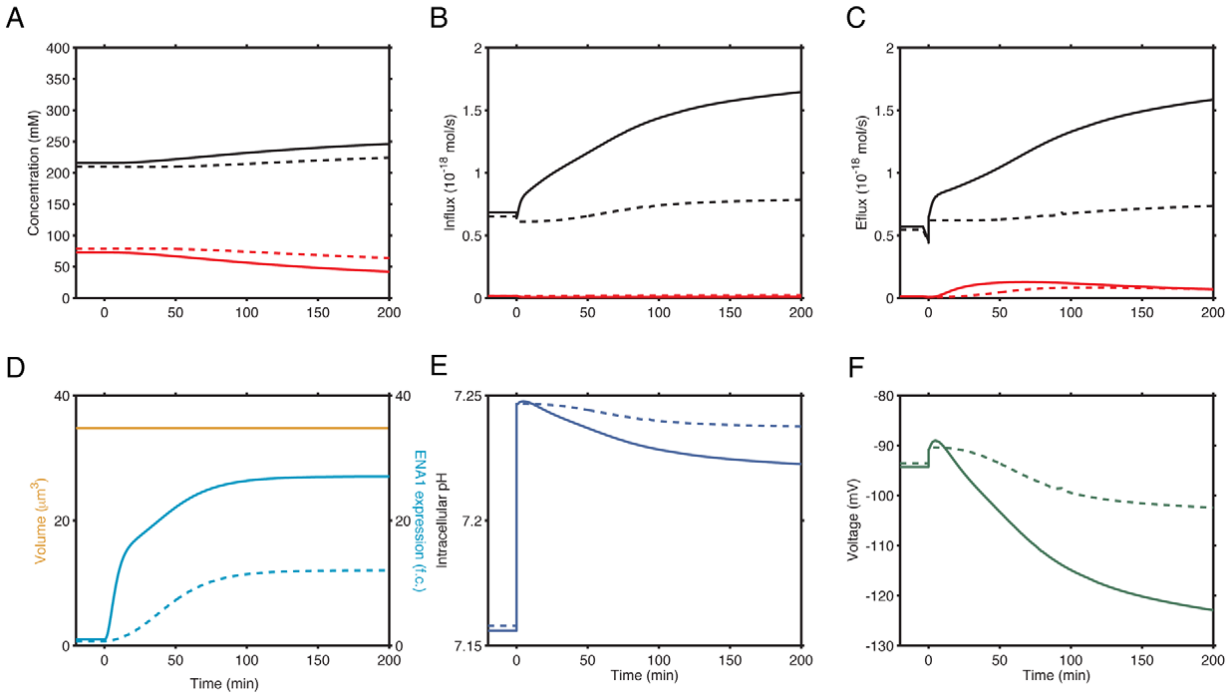
Cellular physiological parameters during time course simulation of alkaline pH 8.0 stress responses in wild type cells with or without the calcineurin inhibitor, FK506. (**A**) Intracellular K^+^ (black lines) and Na^+^ (red lines) concentrations in untreated (solid lines) and FK506 treated cells (dashed lines). (**B**) K^+^ (black lines) and Na^+^ (red lines) influx in untreated (solid lines) and FK506 treated cells (dashed lines). (**C**) K^+^ (black lines) and Na^+^ (red lines) efflux in untreated (solid lines) and FK506 treated cells (dashed lines). (**D**) Cell volume (orange lines) and *ENA1* expression (light blue lines) in wild type cells without (solid lines) and with FK506 (dashed lines). Addition of FK506 does not have any effect on cell volume. (**E**) Intracellular pH in wild type cells without (solid) and with FK506 (dashed). (**F**) Membrane potentials in wild type cells without (solid) and with FK506 (dashed). External baseline Na^+^ and K^+^ concentrations are 5 mM, 1 mM, respectively, and the baseline external pH is 6.5.

We further used this model to investigate the impact of external alkaline pH on intracellular physiological parameters and how cells respond to such changes. Increase in external pH disrupts the H^+^ gradient across the plasma membrane, and therefore, the chemical potential across the plasma membrane is drastically reduced. Our simulation predicts that intracellular pH increased from 7.14 to 7.25 upon pH 8.0 stress (Fig. 7E), due to reductions in the activities of those secondary transporters that utilize the H^+^ gradient, including Nha1p and other H^+^ uptake transporters (Fig. S7). It has been shown that *ENA1* gene expression is highly up-regulated (up to 20 fold), as a result of the activation of several signaling pathways including the calcineurin pathway and the Rim pathway, which negatively act upon Nrg1p [14], [33]. The simulation result agrees with these previous studies and shows that increase in Ena1p activity resulted in an elevated plasma membrane potential (Fig. 7F). The intracellular Na^+^ did not show notable difference in cells treated with and without FK506 (Fig. 7A), suggesting that calcineurin activation is not required for K^+^/Na^+^ homeostasis for alkaline pH adaptation. Since previous work has shown that calcineurin activation is essential for the overall adaptation to alkaline pH stress [34], [35], it is possible that the main function of calcineurin activation in alkaline adaptation is to activate other cellular programs, such as nutrient and cell wall stress responses [34], [35], rather than maintaining K^+^/Na^+^ homeostasis.

### The role of each transporter under stress conditions

To understand the role that each transporter plays in response to different stress conditions, we varied the activity of each transporter in the presence of no stress or four different stresses (NaCl, sorbitol, KCl and alkaline pH).

Varying Pma1p activity had a substantial impact on intracellular pH and membrane potential under all five conditions investigated (Fig. S8), which highlights the importance of this pump in ion homeostasis [36], [37]. In addition to Pma1p, intracellular pH was also sensitive to Nha1p activity except under alkaline pH stress, which suggests a major role for Nha1p in maintaining intracellular pH. This is consistent with previous experimental work [9].

The Trk system was identified as the other transporter with a significant impact on membrane potential, intracellular pH and Na^+^/K^+^ ratio under all five conditions (Fig. S8). Surprisingly, increases in the activity of the Trk system resulted in an increase in intracellular Na^+^/K^+^ ratio in the model under Na^+^ abundant conditions, which contradicts its role of discriminating against Na^+^ uptake. The reason is that in the model, the increase in the activity of Trk system depolarizes the plasma membrane, and thus, triggers K^+^ efflux through Tok1p. Since both K^+^ and Na^+^ enter the cell via the Trk system and only K^+^ is pumped out through Tok1p, higher activity of Trk system causes Na^+^ to accumulate. Hence, this result is likely due to the over-simplified representation of the Trk system in describing the ratio of K^+^/Na^+^ influxes in our model. Further experiments examining the response characteristics of the Trk transporter system are needed to better describe this transporter. Despite this shortcoming, our conclusions of the role of Nha1p, Tok1p, Ena1p, the HOG and the calcineurin pathways are robust to changes in the sub-module describing the Trk system. Because these conclusions depend on the overall dynamics of the intracellular Na^+^/K^+^, which are well described in our integrative model, rather than the ratio of K^+^ and Na^+^ fluxes through the Trk system.

## Discussion

Ion regulation is fundamental to physiology and function across eukaryotic cells. Diverse ion transporters are tightly regulated in a cell to accomplish cellular function [5], [20], [38]. In this study, we have developed an integrative model for ion regulation in budding yeast. Using this model, we investigated the system level properties and predict the role of individual components in the highly coordinated cellular adaptation responses to NaCl, sorbitol, KCl and alkaline pH stress conditions. In particular, we have shown how the Na^+^/K^+^ exporters (Nha1p, Ena1p and Tok1p), the HOG pathway and the calcineurin pathway are coordinated to maintain fundamental physiological parameters, such as K^+^/Na^+^ concentration, the plasma membrane potential and intracellular pH under diverse stress conditions.

High (>0.2 M) external NaCl imposes hyper-osmotic stress as well as Na^+^ ionic stress, while high external sorbitol only imposes hyper-osmotic stress. Our results suggest that the immediate cellular response to NaCl stress and sorbitol stress are similar. High external osmolarity leads to drastic decreases in cell volume and consequently an increase in Na^+^ concentration. In addition to restoring cell volume, an important aspect of immediate adaptation is to decrease intracellular Na^+^. This is mediated by the phosphorylation of Hog1p (P-Hog1p), and its interaction with Nha1p and Top1p at the plasma membrane [13]. Our model analysis predicts that, P-Hog1p inhibits Tok1p activity and this inhibition leads to a depolarized plasma membrane and thus a lower rate of Na^+^ influx. We speculate that the membrane depolarization mediated by the HOG pathway has important implications at the onset of stress when the nature of the external challenge is unknown to the cell: a depolarized membrane leads to reduced activities of secondary transporters and consequently the molecular import of the cell. In cases where the cell is challenged by osmotic stress induced by toxic compounds, membrane depolarization serves as a protective response to prevent uptake of these toxic compounds. This would allow the cell to survive the challenge and respond to the particular stress condition properly through transcriptional regulatory programs.

The model simulations showed marked differences in the intracellular Na^+^ concentration during the long-term adaptations to NaCl stress and sorbitol stress. Under sorbitol stress, intracellular Na^+^ concentration returned to unstressed levels after cell volume is restored, and activation of calcineurin and increased expression of *ENA1* are not required. In contrast, maintaining a high level of Na^+^ extrusion is required throughout the adaptive response to NaCl, which is achieved through the high activity of Nha1p during the initial response to NaCl stress, and upergulation of *ENA1* during the long term response. Although the importance of calcineurin activation and Ena1p upregulation under NaCl stress has been known previously [9], [26], it remains unclear why yeast cells switch transporter use from Nha1p to Ena1p during the adaptation. Here we show that high level of Nha1p activity may lead to a decreased intracellular pH (Fig. S8), which may in turn disrupt proper cellular functions. Therefore, a plausible explanation of the essential role of Ena1p under stress conditions is that it maintains a nontoxic Na^+^ concentration without altering intracellular pH in the cell during the adaptation, at the cost of consuming more cellular energy through ATP hydrolysis. This is consistent with the hypothesis that the high activity of Nha1p at the beginning allows the cell to survive the stress by rapidly reducing Na^+^, while upregulation of Ena1p enables the cell to adapt to the stress condition and restore normal cellular processes in the new environment [13].

The results above suggest that, by restoring the cell volume and enhancing Na^+^ extrusion, the HOG pathway plays essential roles during the immediate adaptation to both osmotic and NaCl stress, whereas the activation of calcineurin pathway is critical for maintaining a low intracellular Na^+^ concentration during the long-term adaptation to NaCl stress alone. Our model provides possible mechanistic explanations underpinning the immediate and long-term adaptation to NaCl stress and osmotic stress.

By simulating the model under KCl stress in a neutral environment and under alkaline pH stress, we found that upregulation of *ENA1* led to notable increases in the plasma membrane potential. In acidic environments, yeast cells make use of the proton gradient created by the Pma1p H^+^-ATPase as an energy source for nutrient uptake [36], [39]. An increase in the external pH disrupts this proton gradient and reduces the solubility of a variety of nutrients. It is likely that the membrane potential becomes an important energy source in these environments. Indeed, it has been shown that several electrogenic transporters that use this electro-gradient as an energy source are up-regulated in alkaline conditions [33], [35]. Therefore, it is possible that, in addition to pumping out toxic ions [40], Ena1p also plays important roles in establishing the membrane potential, thereby maintaining normal uptake of nutrients through secondary transporters under stress conditions.

*ENA1* is transcriptionally upregulated under a diverse stress conditions although why this is the case has not been clearly defined [12]. Here, we show that this upregulation allows the cell to adapt to more extreme conditions either by extruding excessive toxic ions (in the case of NaCl stress) or by re-establishing membrane potential (in the case of KCl and alkaline pH stresses). The consequence of this upregulation is the consumption of the energy produced through cellular metabolism via ATP hydrolysis. In contrast, the production of Ena1p is kept at a low level under unstressed conditions where Tok1p and Nha1p are used to maintain monovalent ion homeostasis without consuming the energy stored in ATP. We speculate this may represent a strategy used by the cell to maximize energy use under different conditions.

One question concerning the system level property of the ion regulation process is that why yeast cells use a wide range of transporters to regulate a relatively few monovalent ions, i.e. K^+^, Na^+^ and H^+^. In this study, we have shown that, by regulating different subsets of these transporters under different stress conditions, yeast cell is able to utilize the energy available to the cell in the environment and maintain fundamental physiological parameters within a narrow range. Therefore, the diverse ion transporters in yeast cells may reflect the different strategies that yeast cells employed to respond and adapt to the ever-changing environment.

One limitation of our integrated model is the uncertainties of the parameter values, which is an inherent problem with any large-scale model. This limitation is partly addressed by constructing and validating the model on experimental data and by analyzing the sensitivity of the system to perturbations in the activities of the transporters. However, the parameters estimated in this study may not be an accurate approximation for all cellular components due to limited availability of experimental data. For example, inconsistency between experimental data and the model prediction of the Trk system exists and the Ca^2+^ signaling is highly simplified in the model. Although these shortcomings in the model should not impact the our conclusions qualitatively, more systematic experimental studies using standardized protocols are needed to measure the characteristics of each cellular component under different conditions (for example, see [41]), in order to make more precise quantitative predictions. Therefore, the model presented here should not be taken as an exact quantitative representation of the cellular adaption processes to ionic stresses. Rather, it is a theoretical framework for qualitatively examining the overall system property and the role of each component, and to generate rational hypotheses for further experimental validation.

In this study, we have constructed an integrated model to understand the system level properties of the coordinated responses of cellular components to ionic stresses. This model provides a suitable tool for the study of transporter activity, ion regulation and homeostasis in yeast, although it is by no means complete. The vacuole is known to play an important role in adaptation to NaCl stress and maintaining intracellular pH [30], [42]. The process of Ca^2+^ homeostasis is described in a simplistic way in this model. Therefore, future work should focus on extending the model to include the vacuolar compartment/transporters and Ca^2+^ transporters at both the plasma membrane and vacuolar membrane. Since monovalent ion regulation and transporter activities have been shown to be related to cell cycle progression [6], a variety of diseases [43], toxin effects [11], and the membrane potential is an important determinant of drug uptake [44], we hope this model will also prove a useful tool in understanding these processes. Furthermore, since the underlying principles of monovalent ion regulations are similar in plant cells and mammalian cells [45], [46], [47], this integrated model can be easily adapted to study the role of transporters, regulatory molecules and drug effects in plant and mammalian systems.

## Methods

### Transporter module

In general, we model the activity of each transporter using biochemical equations that derived from the chemical reaction mediated by the transporter, the Gibbs energy conservation equation or equations describing the flow of ions [48]. The equation and parameters for each transporter is then modified such that the response characteristics of the transporter are consistent with previously published data. As an example, we derive the equation describing the H^+^ fluxes through the H^+^-ATPase, Pma1p, for a given intracellular and extracellular H^+^ concentration below. See Text S1 for the derivations for other transporters.

Pma1p transports H^+^ across the plasma membrane using the energy released from ATP hydrolysis. The chemical reaction for Pma1p-driven H^+^ extrusion is:
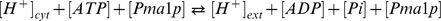
Then, the flux through Pma1p can be approximated as [48]:
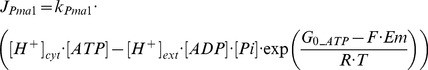
(1)where *k_Pma1_* is a rate constant, *[ATP]*, *[ADP]* and *[Pi]* are the concentrations of intracellular ATP, ADP and phosphate, respectively, *G_0_ATP_* is the free energy released by ATP hydrolysis, *R* is the gas constant, *T* is the absolute temperature, *F* is the Faraday constant and *Em* is the membrane potential. The value of *k_Pma1_* can be estimated from the experimentally measured Pma1p activity in [7].

After deriving the transporter activities, we calculate the changes in intracellular cation concentration by combining the fluxes across the plasma membrane through transporters and diffusion processes and cellular metabolism. We assume that cations are well mixed in the cell. We do not consider the vacuole (or other cellular compartment) explicitly, although it has been shown that the vacuole plays roles in regulating cytosolic cation concentration [30]. The reasons are that the transporters at the membrane of the vacuole are not well characterized, and the main intracellular cation regulation is achieved by regulating flux through the plasma membrane [5]. Incorporating the impact of vacuolar regulation on the cytosolic cation concentrations will be an extension of the model in future work.

The equations are in the following general form:
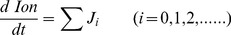
(2)where *Ion* is the total amount of an ion in a cell, *J_i_* describes the rates of flux of the i^th^ ion as a result of transporters activities, passive diffusion across the plasma membrane and cellular metabolism.

Post-translational regulations of cation transporters are assumed to be fast reactions, i.e. reactions are always at equilibrium. This is a reasonable approximation since the rate of post-translation modulation is usually measured in seconds, while the transcriptional response can take 10–30 minutes. Each regulatory protein acts as an enzyme and the distribution of the transporters in different states is in equilibrium can be described as:
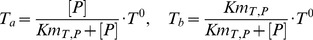
(3)where *T_a_, T_b_* are the amount of a transporter in state a and b, respectively, *T^0^* is the total amount of the transporter. *P* is the regulatory protein, and *Km_T,P_* is the ratio of the backward reaction over the forward reaction.

As an example, we briefly derive the equations describing the post-translational regulation of Nha1p by Hog1p here. Interaction of Hog1p and Nha1p changes Nha1p from the unphosphorylated state (*Nha1_unphos_*) to the phosphorylated state (*Nha1_phos_*). Then, the amount of Nha1p in the phosphorylated state and the un-phosphorylated state for a given Hog1p concentration in the cytosol (Hog1PP_cyt_) can be approximated as:
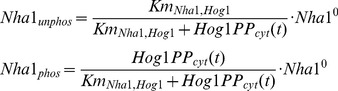
(4)where *Km_Nha1,Hog1_* is the dissociation constant for Hog1p regulation and Nha1^0^ is the total amount of Nha1p.

### Signaling module

We describe the activities of the HOG pathway, the calcineurin pathway, and Nrg1p and their transcriptional regulation of *ENA1* in the transcriptional regulation module. This is modeled using combinations of mass action and Hill-type equations. The ODEs are in the following form:
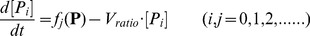
(5)where *[P_i_]* is the concentration of the *i* ^th^ protein or protein complex, ***P*** is the concentration vector of all protein and protein complexes, *f_j_* is a regulatory function in the form of mass action and Hill-type functions, and *V_ratio_* is a time dependent rate that describes the dependence of the changes of the protein concentration in the cytosol on the changes in the cytosol volume [17] (see ‘The HOG pathway’ section in Text S1 for details). Basically, the first term at the right hand side of equation (5) describes the changes in the protein concentration due to chemical reactions, whereas the second term describes the changes in the protein concentration due to changes in volume.

### Volume module

We consider the cell as a semi-permeable compartment that responds to changes in external osmolarity by changing volume. The rate of change in cell volume is proportional to the rate of water flow, which is dependent on the pressure difference across the plasma membrane. Change in volume is then given by:
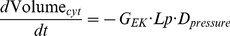
(6)where *G_EK_* is a geometrical factor relating the volume of a sphere to its surface, *Lp* is the hydraulic membrane permeability and *D_Pressure_* is the pressure difference between external pressure plus turgor pressure and the interior pressure of the cell plasma membrane.

### Integration

These three modules were integrated to describe the overall process of ion regulation. The rates of change in the levels of Na^+^, K^+^ and H^+^ are determined by the fluxes through the relevant transporters. Levels of signaling molecules determine the activities of those transporters as well as the levels of activated Hog1p, calcineurin and Nrg1p. At the transcription level, activation of the HOG pathway, the calcineurin pathway and Nrg1p determine the expression of *ENA1*. All together, this integrative model consists of 23 state variables and 123 parameters. The ODEs can be found in Table S1. The parameter values in the model and the initial conditions are presented in Table S2, S3, S4, S5.

### Modeling mutants

See Text S1 for the changes to the model to simulate the mutant phenotypes in this study.

### Sensitivity analysis

To determine how each sub-module is constrained by the data that was chosen for model integration and validation, we performed sensitivity analysis using Latin-Hypercube Sampling (LHS) [49]. Specifically, we have performed sensitivity analysis for the parameters that describe the total flux of the major transporters, the activations of the HOG pathway and the calcineurin pathway and the upregulation of *ENA1* expression (see Table S6). The values of these parameters were sampled from a log-uniform distribution with the 50% to 200% of their original values as the lower and upper bounds, respectively. A total of 10,000 samples of parameter sets were drawn. The linear least-square errors between simulation results and data points were estimated. The partial rank correlation coefficients were calculated based on the parameter samples and the resulting errors. The results are shown in Table S6.

## Supporting Information

Figure S1 **Workflow of the construction of the integrated model.** The integrated model consists of three modules: the ‘transporter’ module, the ‘signaling’ module and the ‘volume’ model. These three modules were composed of several sub-modules, which were first constructed based on previously published models or data sets. These sub-modules are then linked together into an integrated model. This integrated model is then compared and validated by published data sets that were not used during model construction. Finally, predictions are made from the integrated model.
(TIF)

Figure S2 **Transporter activities and enzyme concentrations during time course simulation of 0.8M NaCl stress responses in wild type cells with or without the calcineurin inhibitor, FK506.** Fluxes through transporters in panels **A–K** are in the unit of 1e-18 mol/s. Concentrations of enzymes in panels **L–O** are in the unit of mM.
(TIF)

Figure S3 **Cellular physiological parameters during time course simulation of 1.6M sorbitol stress responses in wild type cells with or without the calcineurin inhibitor, FK506.** (**A**) Intracellular K^+^ (black lines) and Na^+^ (red lines) concentrations in untreated (solid lines) and FK506 treated cells (dashed lines). (**B**) K^+^ (black lines) and Na^+^ (red lines) influxes in untreated (solid lines) and FK506 treated cells (dashed lines). (**C**) K^+^ (black lines) and Na^+^ (red lines) effluxes in untreated (solid lines) and FK506 treated cells (dashed lines). (**D**) Cell volume (orange lines) and *ENA1* expression (light blue lines) in wild type cells without (solid lines) and with FK506 (dashed lines). Addition of FK506 does not have any effect on cell volume. (**E**) Intracellular pH in wild type cells without (solid blue lines) and with FK506 (dashed blue lines). (**F**) Membrane potentials in wild type cells without (solid green lines) and with FK506 (dashed green lines).
(TIF)

Figure S4 **Transporter activities and enzyme concentrations during time course simulation of 1.6M sorbitol stress responses in wild type cells with or without the calcineurin inhibitor, FK506.** Fluxes through transporters in panels **A–K** are in the unit of 1e-18 mol/s. Concentrations of enzymes in panels **L–O** are in the unit of mM.
(TIF)

Figure S5 **Cellular physiological parameters during time course simulation of 0.8M KCl stress responses in wild type cells with or without the calcineurin inhibitor, FK506.** (**A**) Intracellular K^+^ (black lines) and Na^+^ (red lines) concentrations in untreated (solid lines) and FK506 treated cells (dashed lines). (**B**) K^+^ (black lines) and Na^+^ (red lines) influxes in untreated (solid lines) and FK506 treated cells (dashed lines). (**C**) K^+^ (black lines) and Na^+^ (red lines) effluxes in untreated (solid lines) and FK506 treated cells (dashed lines). (**D**) Cell volume (orange lines) and *ENA1* expression (light blue lines) in wild type cells without (solid lines) and with FK506 (dashed lines). Addition of FK506 does not have any effect on cell volume. (**E**) Intracellular pH in wild type cells without (solid blue lines) and with FK506 (dashed blue lines). (**F**) Membrane potentials in wild type cells without (solid green lines) and with FK506 (dashed green lines).
(TIF)

Figure S6 **Transporter activities and enzyme concentrations during time course simulation of 0.8M KCl stress responses in wild type cells with or without the calcineurin inhibitor, FK506.** Fluxes through transporters in panels **A–K** are in the unit of 1e-18 mol/s. Concentrations of enzymes in panels **L–O** are in the unit of mM.
(TIF).

Figure S7 **Transporter activities and enzyme concentrations during time course simulation of alkaline pH 8.0 stress responses in wild type cells with or without the calcineurin inhibitor, FK506.** Fluxes through transporters in panels **A–K** are in the unit of 1e-18 mol/s. Concentrations of enzymes in panels **L–O** are in the unit of mM.
(TIF)

Figure S8 **Sensitivity of membrane potential, intracellular pH and intracellular Na^+^/K^+^ ratio to the variation in each pump under unstressed, NaCl, osmotic, KCl and alkaline pH stress conditions.** The sensitivities of the membrane potential to changes in transport activity are shown in panels (A–E). The sensitivities of the intracellular pH are shown in panels (F–J). The sensitivities of the membrane potential are shown in panels (K–O). Five environmental conditions are considered: unstressed condition (panels (A,F,K)), NaCl stress (panels (B,G,L)), osmotic stress (panels (C,H,M)), KCl stress (panels (D,I,N)) and alkaline pH stress (panels (E,J,O)), The activity of each pump is varied as fold changes (x-axis) while the activities of other pumps are kept unchanged. The steady state values are shown on y-axis. Figure legend indicates the transporter varied.
(TIF)

Table S1 **ODE system for the integrative model.**
(PDF)

Table S2 **Parameters for the transporter module.**
(PDF)

Table S3 **Parameters for the signaling module.**
(PDF)

Table S4 **Basic physiological parameters.**
(PDF)

Table S5 **Initial concentrations for the ODE system.**
(PDF)

Table S6 **Parameter sensitivity to experimental data using Latin-Hypercube Sampling.**
(PDF)

Text S1 **Supplementary material.**
(PDF)
