# Supplementary figures and images for: An Integrative Model of Ion Regulation in Yeast
Source: PLoS Comput Biol. 2013 Jan 17;9(1):e1002879. doi: 10.1371/journal.pcbi.1002879 (PMC3547829; doi:10.1371/journal.pcbi.1002879)

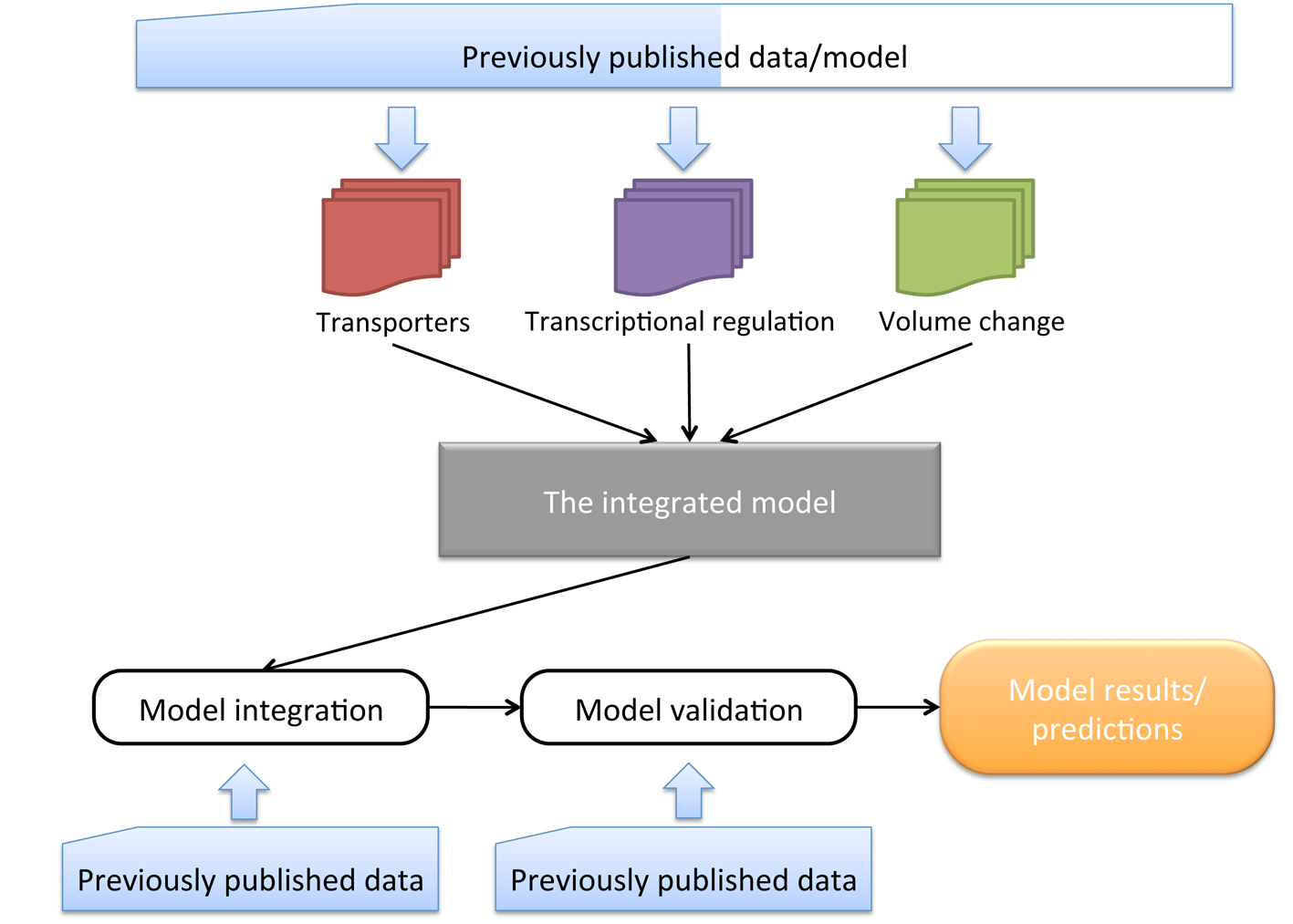

Supplement: Figure S1 — Workflow of the construction of the integrated model. The integrated model consists of three modules: the ‘transporter’ module, the ‘signaling’ module and the ‘volume’ model. These three modules were composed of several sub-modules, which were first constructed based on previously published models or data sets. These sub-modules are then linked together into an integrated model. This integrated model is then compared and validated by published data sets that were not used during model construction. Finally, predictions are made from the integrated model. (TIF) [file pcbi.1002879.s001.tif]

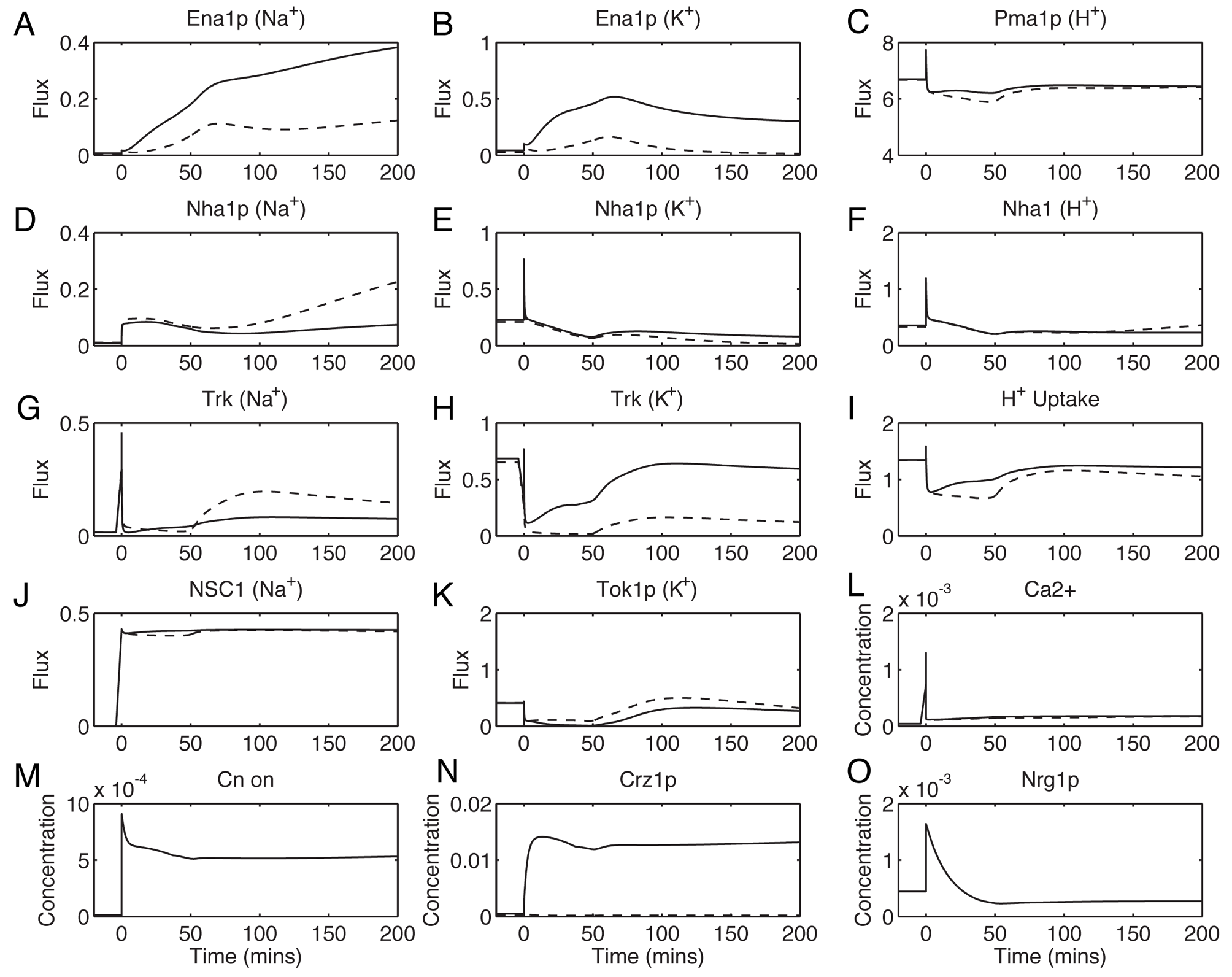

Supplement: Figure S2 — Transporter activities and enzyme concentrations during time course simulation of 0.8M NaCl stress responses in wild type cells with or without the calcineurin inhibitor, FK506. Fluxes through transporters in panels A–K are in the unit of 1e-18 mol/s. Concentrations of enzymes in panels L–O are in the unit of mM. (TIF) [file pcbi.1002879.s002.tif]

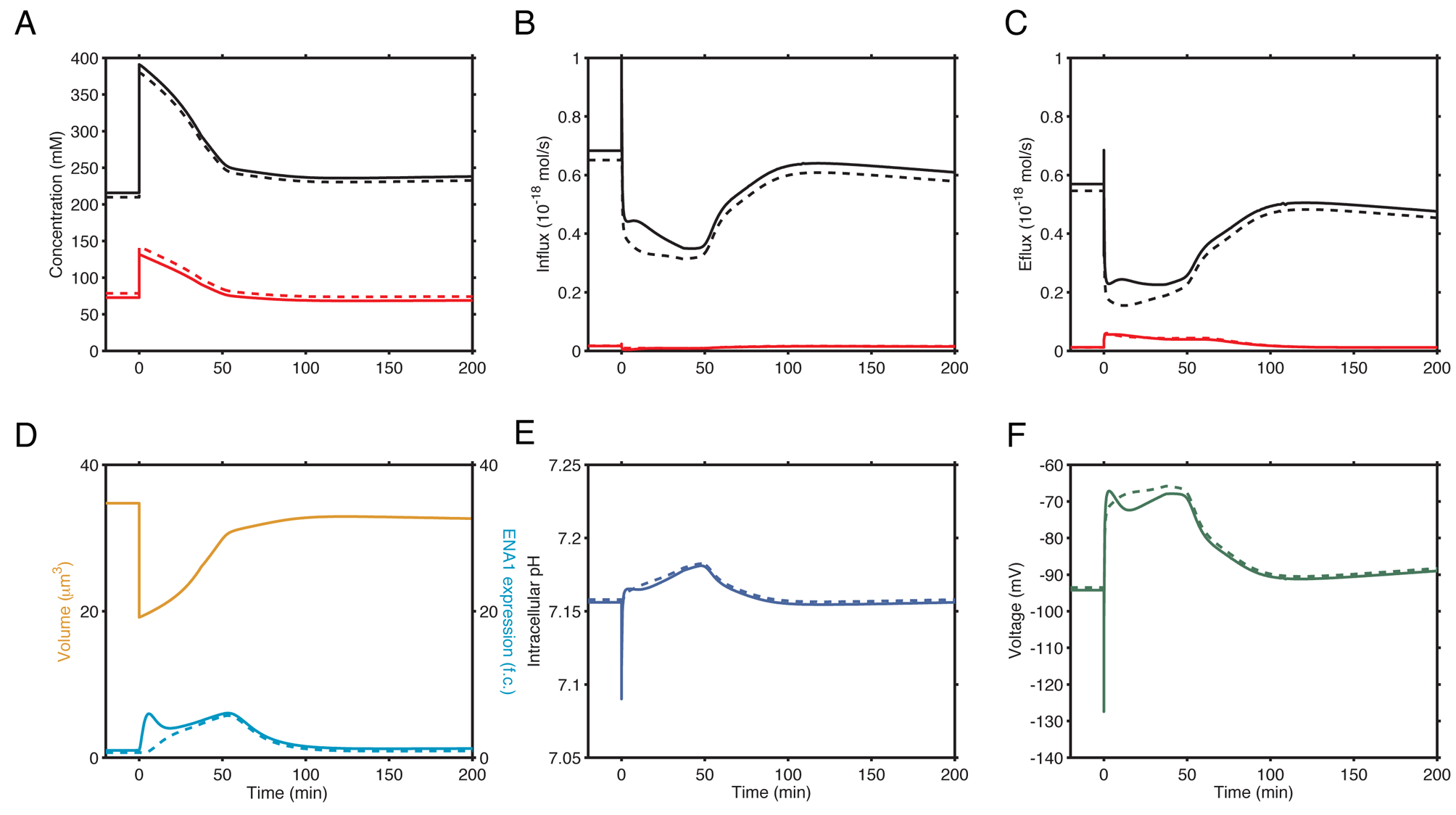

Supplement: Figure S3 — Cellular physiological parameters during time course simulation of 1.6M sorbitol stress responses in wild type cells with or without the calcineurin inhibitor, FK506. (A) Intracellular K+ (black lines) and Na+ (red lines) concentrations in untreated (solid lines) and FK506 treated cells (dashed lines). (B) K+ (black lines) and Na+ (red lines) influxes in untreated (solid lines) and FK506 treated cells (dashed lines). (C) K+ (black lines) and Na+ (red lines) effluxes in untreated (solid lines) and FK506 treated cells (dashed lines). (D) Cell volume (orange lines) and ENA1 expression (light blue lines) in wild type cells without (solid lines) and with FK506 (dashed lines). Addition of FK506 does not have any effect on cell volume. (E) Intracellular pH in wild type cells without (solid blue lines) and with FK506 (dashed blue lines). (F) Membrane potentials in wild type cells without (solid green lines) and with FK506 (dashed green lines). (TIF) [file pcbi.1002879.s003.tif]

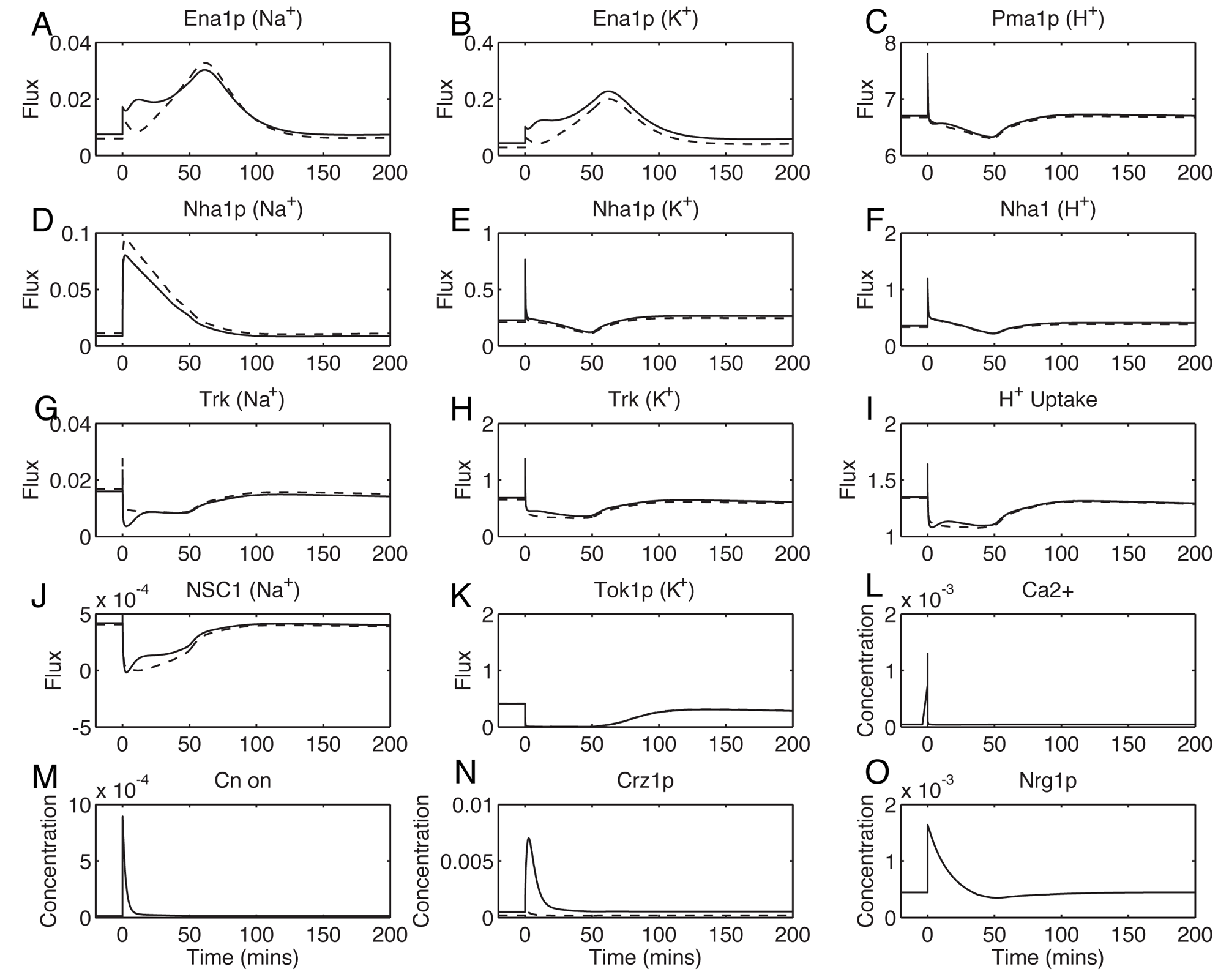

Supplement: Figure S4 — Transporter activities and enzyme concentrations during time course simulation of 1.6M sorbitol stress responses in wild type cells with or without the calcineurin inhibitor, FK506. Fluxes through transporters in panels A–K are in the unit of 1e-18 mol/s. Concentrations of enzymes in panels L–O are in the unit of mM. (TIF) [file pcbi.1002879.s004.tif]

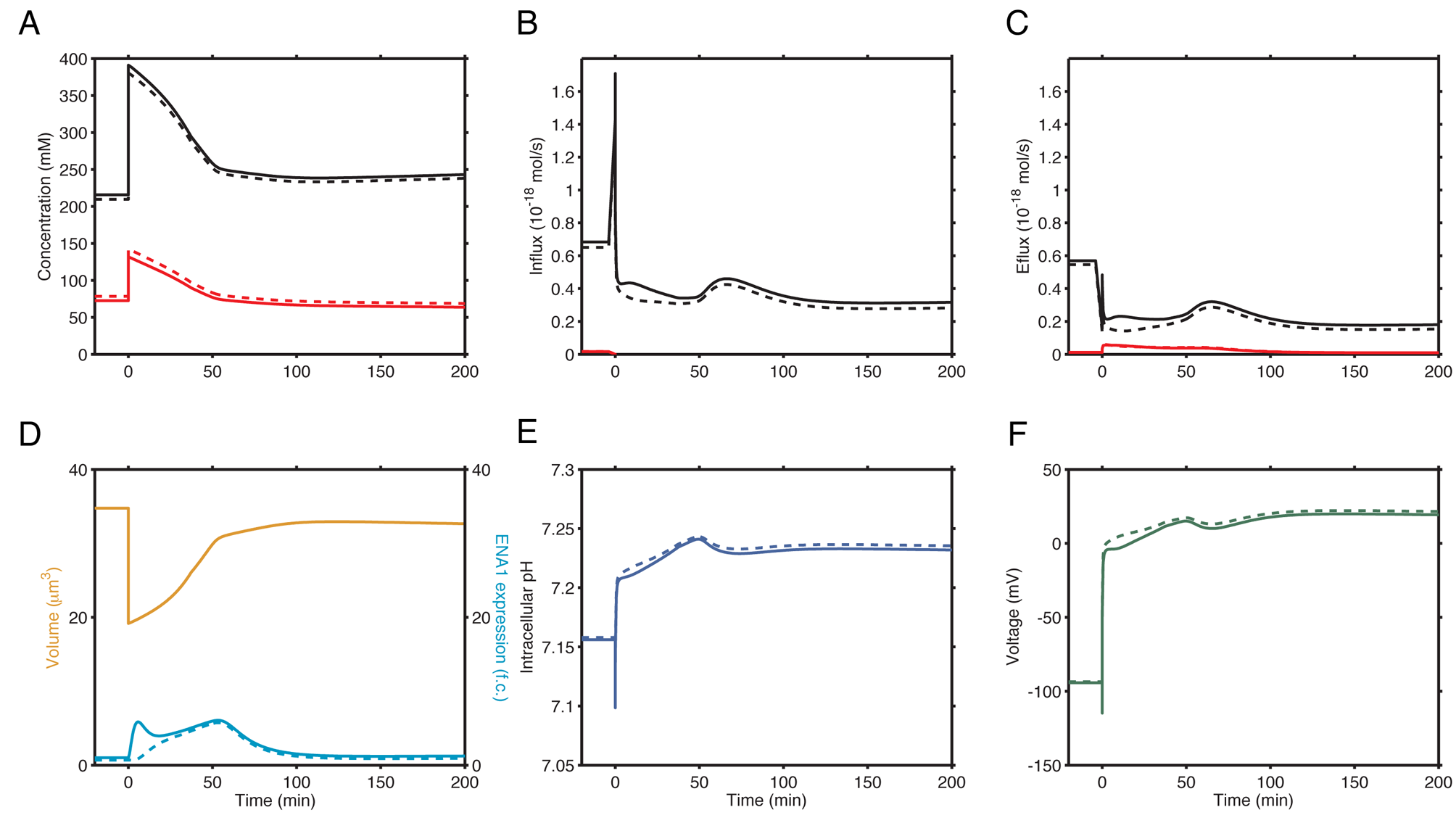

Supplement: Figure S5 — Cellular physiological parameters during time course simulation of 0.8M KCl stress responses in wild type cells with or without the calcineurin inhibitor, FK506. (A) Intracellular K+ (black lines) and Na+ (red lines) concentrations in untreated (solid lines) and FK506 treated cells (dashed lines). (B) K+ (black lines) and Na+ (red lines) influxes in untreated (solid lines) and FK506 treated cells (dashed lines). (C) K+ (black lines) and Na+ (red lines) effluxes in untreated (solid lines) and FK506 treated cells (dashed lines). (D) Cell volume (orange lines) and ENA1 expression (light blue lines) in wild type cells without (solid lines) and with FK506 (dashed lines). Addition of FK506 does not have any effect on cell volume. (E) Intracellular pH in wild type cells without (solid blue lines) and with FK506 (dashed blue lines). (F) Membrane potentials in wild type cells without (solid green lines) and with FK506 (dashed green lines). (TIF) [file pcbi.1002879.s005.tif]

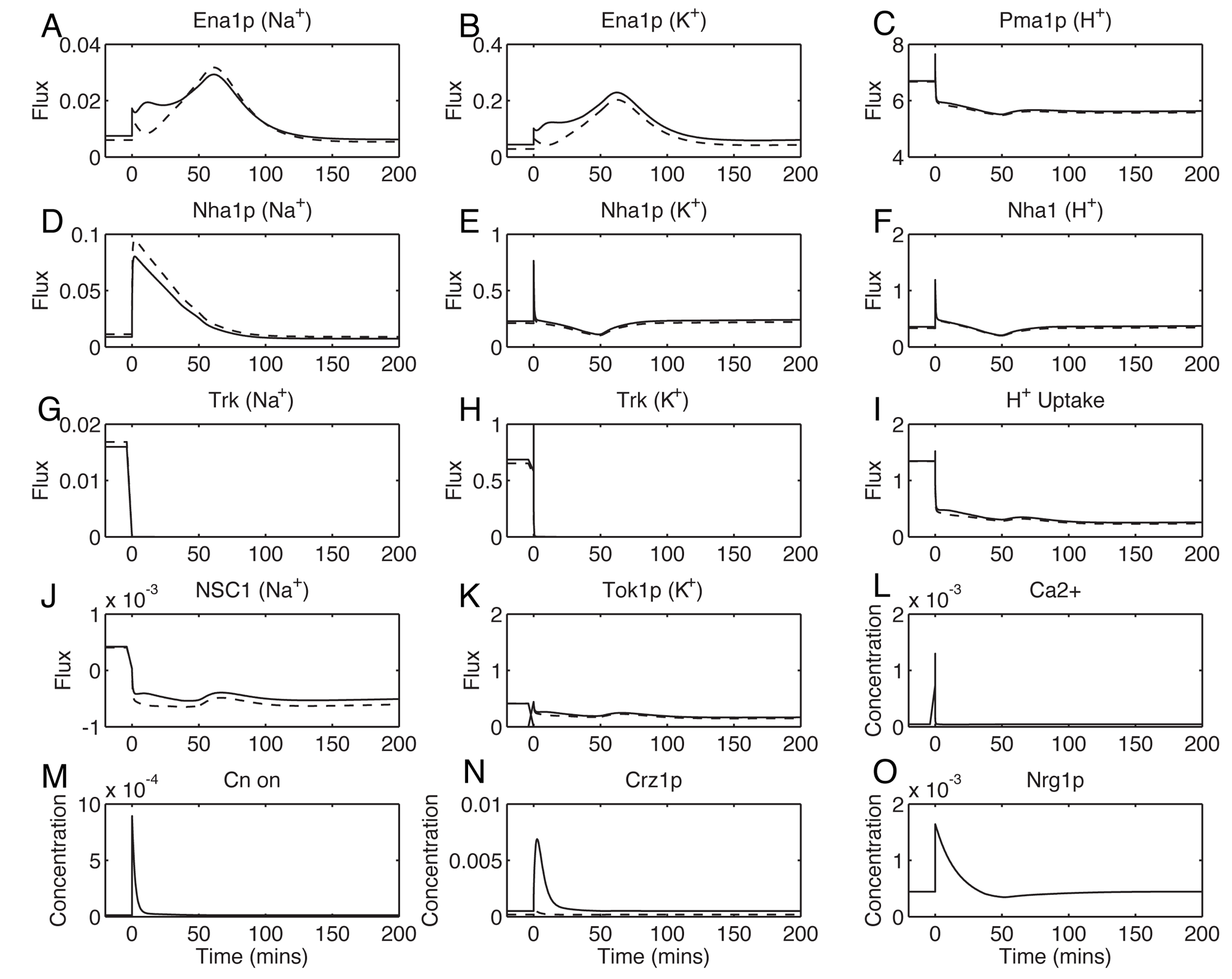

Supplement: Figure S6 — Transporter activities and enzyme concentrations during time course simulation of 0.8M KCl stress responses in wild type cells with or without the calcineurin inhibitor, FK506. Fluxes through transporters in panels A–K are in the unit of 1e-18 mol/s. Concentrations of enzymes in panels L–O are in the unit of mM. (TIF). [file pcbi.1002879.s006.tif]

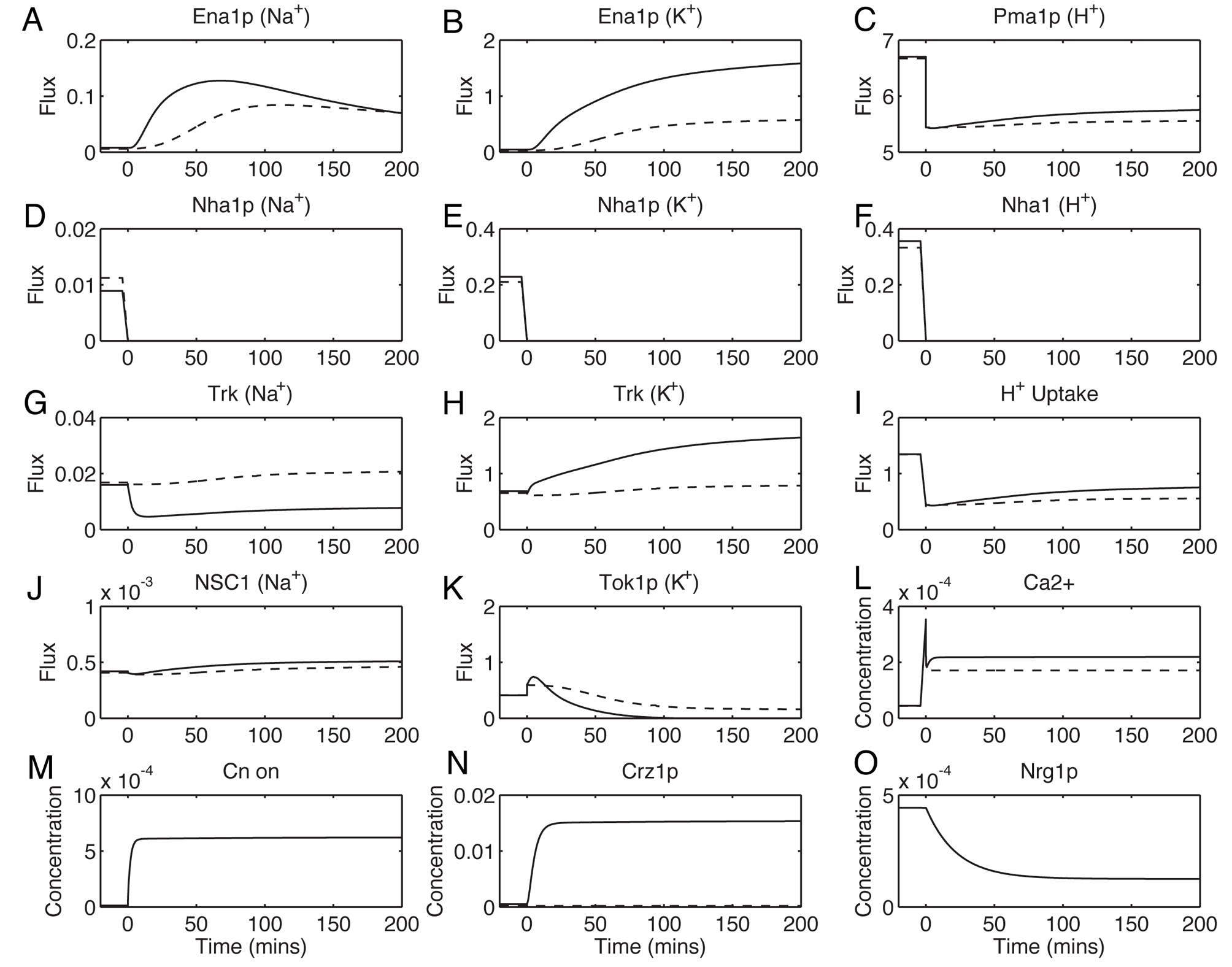

Supplement: Figure S7 — Transporter activities and enzyme concentrations during time course simulation of alkaline pH 8.0 stress responses in wild type cells with or without the calcineurin inhibitor, FK506. Fluxes through transporters in panels A–K are in the unit of 1e-18 mol/s. Concentrations of enzymes in panels L–O are in the unit of mM. (TIF) [file pcbi.1002879.s007.tif]

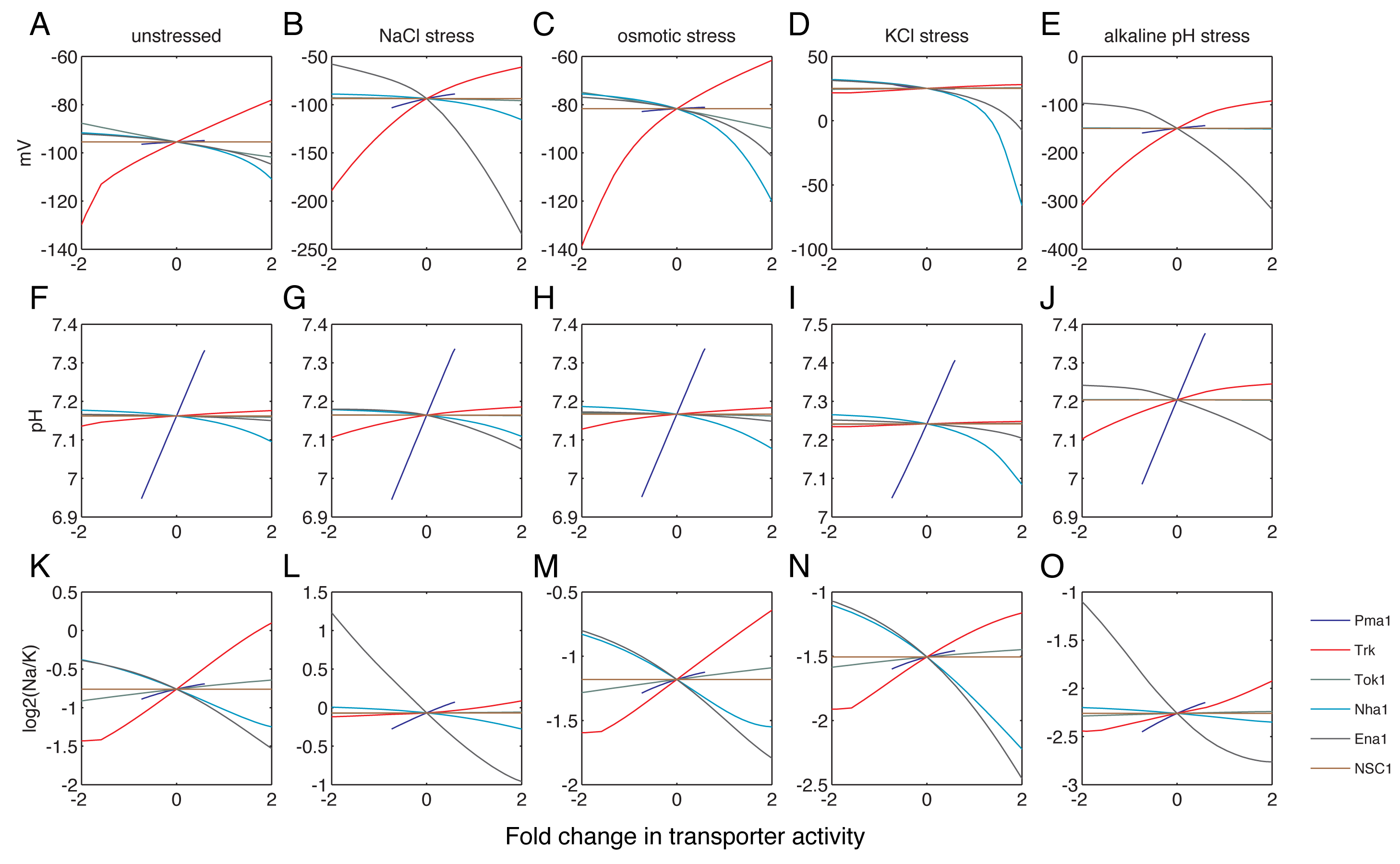

Supplement: Figure S8 — Sensitivity of membrane potential, intracellular pH and intracellular Na+/K+ ratio to the variation in each pump under unstressed, NaCl, osmotic, KCl and alkaline pH stress conditions. The sensitivities of the membrane potential to changes in transport activity are shown in panels (A–E). The sensitivities of the intracellular pH are shown in panels (F–J). The sensitivities of the membrane potential are shown in panels (K–O). Five environmental conditions are considered: unstressed condition (panels (A,F,K)), NaCl stress (panels (B,G,L)), osmotic stress (panels (C,H,M)), KCl stress (panels (D,I,N)) and alkaline pH stress (panels (E,J,O)), The activity of each pump is varied as fold changes (x-axis) while the activities of other pumps are kept unchanged. The steady state values are shown on y-axis. Figure legend indicates the transporter varied. (TIF) [file pcbi.1002879.s008.tif]
