# Supplementary material for: An Integrative Model of Ion Regulation in Yeast
Source: PLoS Comput Biol. 2013 Jan 17;9(1):e1002879. doi: 10.1371/journal.pcbi.1002879 (PMC3547829; doi:10.1371/journal.pcbi.1002879)
Supplement: Table S1 — ODE system for the integrative model. (PDF) [file pcbi.1002879.s009.pdf]

Table S1. ODE system for the integrative model*

$$\frac{d\,\mathrm{H}^+}{dt} = -J_{Pma1} + \frac{3}{2}\cdot J_{Nha1,Na} + \frac{3}{2}\cdot J_{Nha1,K} + J_{H\_uptake} + J_{H\_production}$$

$$\frac{d\,Na^+}{dt} = -J_{Ena1,Na} - J_{Nha1,Na} + J_{Trk,Na} + J_{NSC1,Na} + J_{diff,Na}$$

$$\frac{d\,K^+}{dt} = -J_{Ena1,K} - J_{Nha1,K} + J_{Trk1,K} + J_{NSC1,K} - J_{Tok1} + J_{diff,K}$$

$$\frac{d[Pbs2]}{dt} = -\frac{K_{pho}^{Pbs2}\cdot[Pbs2]}{1+\left(\frac{P_{tur}}{\alpha_{Hog1}}\right)^8} + K_{depho}^{Pbs2}\cdot[Pbs2PP] - [Pbs2]\cdot V_{ratio}$$

$$\frac{d[Pbs2PP]}{dt} = \frac{K_{pho}^{Pbs2}\cdot[Pbs2]}{1+\left(\frac{P_{tur}}{\alpha_{Hog1}}\right)^8} - K_{depho}^{Pbs2}\cdot[Pbs2PP] - [Pbs2PP]\cdot V_{ratio}$$

$$\frac{d[Hog1c]}{dt} = -K_{pho}^{Hog1}\cdot[Pbs2PP]\cdot[Hog1c] + K_{depho}^{Hog1PPc}\cdot[Hog1PPc] - K_{imp}^{Hog1c}\cdot[Hog1c] + \frac{K_{exp}^{Hog1n}\cdot[Hog1n]\cdot V_{nuc}}{V_{cyt}} - [Hog1c]\cdot V_{ratio}$$

$$\frac{d[Hog1PPc]}{dt} = K_{pho}^{Hog1}\cdot[Pbs2PP]\cdot[Hog1c] - K_{depho}^{Hog1PPc}\cdot[Hog1PPc] - K_{imp}^{Hog1PPc}\cdot[Hog1PPc] + \frac{K_{exp}^{Hog1PPn}\cdot[Hog1PPn]\cdot V_{nuc}}{V_{cyt}} - [Hog1PPc]\cdot V_{ratio}$$

$$\frac{d[Hog1n]}{dt} = \frac{K_{imp}^{Hog1c}\cdot[Hog1c]\cdot V_{cyt}}{V_{nuc}} - K_{exp}^{Hog1n}\cdot[Hog1n] + K_{depho}^{Hog1PPn}\cdot[Hog1PPn] - [Hog1n]\cdot V_{ratio}$$

$$\frac{d[Hog1PPn]}{dt} = \frac{K_{imp}^{Hog1PPc}\cdot[Hog1PPc]\cdot V_{cyt}}{V_{nuc}} - K_{exp}^{Hog1PPn}\cdot[Hog1PPn] - K_{depho}^{Hog1PPn}\cdot[Hog1PPn] - [Hog1PPn]\cdot V_{ratio}$$

$$\frac{d[Gly_{\text{int}}]}{dt} = K_{s0}^{Glyc} + \frac{K_{s1}^{Glyc} \cdot (totalHog1PP)^4}{\beta_{Hog1}^4 + (totalHog1PP)^4} + K_{s2}^{Glyc} \cdot [Yt] - \left(K_{exp0}^{Glyc} + \frac{K_{exp1}^{Glyc} \cdot (P_{tur})^{12}}{(\gamma_{Hog1})^{12} + (P_{tur})^{12}} \right) \cdot [Gly_{\text{int}}] - [Gly_{\text{int}}] \cdot V_{ratio}$$

$$\frac{d[Yt]}{dt} = K_{s0}^{Yt} + K_{s1}^{Yt} \cdot [z4] - K_t^{Yt} \cdot [Yt] - [Yt] \cdot V_{ratio}$$

$$\frac{d[z1]}{dt} = \frac{4 \cdot ([Hog1PPn] - [z1])}{\tau}$$

$$\frac{d[z2]}{dt} = \frac{4 \cdot ([z1] - [z2])}{\tau}$$

$$\frac{d[z3]}{dt} = \frac{4 \cdot ([z2] - [z3])}{\tau}$$

$$\frac{d[z4]}{dt} = \frac{4 \cdot ([z3] - [z4])}{\tau}$$

$$\frac{d\ [Ca^{2+}]}{dt} = C_{Ca} - d_{Ca} \cdot [Ca^{2+}] + k_{Ca,cyt} \cdot \frac{[Na^+]_{Cyt}^{h\_Na\_cyt}}{[Na^+]_{Cyt}^{h\_Na\_cyt} + Km_{Ca,cyt}^{h\_Na\_cyt}} + k_{Ca,ext} \cdot \frac{[Na^+]_{ext}^{h\_Na\_ext}}{[Na^+]_{ext}^{h\_Na\_ext} + Km_{Ca,ext}^{h\_Na\_ext}} + k_{Ca,pH} \cdot (pH_{ext} - 6.5) - 3 \cdot k_{CN,a} \cdot [\mathrm{Ca}^{2+}]^3 \cdot [CN_{off}] + 3 \cdot k_{CN,da} \cdot [CN] - [Ca^{2+}] \cdot V_{ratio}$$

$$\frac{d\ [\mathrm{CN_{off}}]}{dt} = -k_{CN,a} \cdot [\mathrm{Ca}^{2+}]^3 \cdot [CN_{off}] + k_{CN,da} \cdot [CN] + k_{CN\_Ppz,da} \cdot [Ppz] \cdot [CN] - [\mathrm{CN_{off}}] \cdot V_{ratio}$$

$$\frac{d\ [\mathrm{CN}]}{dt} = k_{CN,a} \cdot [\mathrm{Ca}^{2+}]^3 \cdot [CN_{off}] - k_{CN,da} \cdot [CN] - k_{CN\_Ppz,da} \cdot [Ppz] \cdot [CN] - [\mathrm{CN}] \cdot V_{ratio}$$

$$\frac{d\ [\mathrm{Crz1}]}{dt} = C_{Crz1} - d_{Crz1} \cdot [\mathrm{Crz1}] + k_{Crz1} \cdot [CN] - [\mathrm{Crz1}] \cdot V_{ratio}$$

$$\frac{d\ [\mathrm{Nrg1}]}{dt} = C_{Nrg1} \cdot \frac{Km_{Nrg1,pH}}{Km_{Nrg1,pH} + k_{pHext}} - d_{Nrg1} \cdot [\mathrm{Nrg1}] - [Nrg1] \cdot V_{ratio}$$

$$\frac{d\ [E\mathrm{NA1_{mRNA}}]}{dt} = C_{ENA1,Nrg1} \cdot \frac{Km_{ENA1,Nrg1}^{h\_ENA1Nrg1}}{Km_{ENA1,Nrg1}^{h\_ENA1Nrg1} + [Nrg1]^{h\_ENA1Nrg1}} + k_{ENA1,Crz1} \cdot [Crz1] + k_{ENA1,Hog1} \cdot [z2] - d_{ENA1mRNA} \cdot [E\mathrm{NA1_{mRNA}}]$$

$$\frac{d\ [E\mathrm{na1}]}{dt} = kt_{Ena1} \cdot [E\mathrm{NA1_{mRNA}}] - d_{Ena1} \cdot [E\mathrm{na1}] - [Ena1] \cdot V_{ratio}$$

$$\frac{d\ \mathrm{Volume_{cyt}}}{dt} = -G_{EK} \cdot Lp \cdot D_{\mathrm{Pr}essure}$$

* For expressions of the transporter activities and volume changes, see Section 2-4 in the Supporting text (Text S1).
