# Supplementary material for: An Integrative Model of Ion Regulation in Yeast
Source: PLoS Comput Biol. 2013 Jan 17;9(1):e1002879. doi: 10.1371/journal.pcbi.1002879 (PMC3547829; doi:10.1371/journal.pcbi.1002879)
Supplement: Table S2 — Parameters for the transporter module. (PDF) [file pcbi.1002879.s010.pdf]

Table S2. Parameters for the transporter module

**Model /** Parameter Name	**Value**	**Unit**	**Reference**
Ena1p			
k_{Ena1}*	1.98*1e-4	1e-18 mol/(s*mM^2)	Estimated from [27]
$Km_{Ena1,Na}$	200	mM	Taken from [58]
$Km_{Ena1,K}$	300	mM	Estimated from [58]
Nha1p			
k_{Nha1}*	1.092*1e+5	1e-18 mol /(s*mM^5)	Estimated from [12]
$Km_{Nha1,Na}$	12.7	mM	Taken from [36]
$Km_{Nha1\_high,K}$	12.4	mM	Taken from [36]
$Km_{Nha1\_low,K}$	1240	mM	
$Km_{Nha1,Hog1}$	1e-4	mM	
Tok1p			
$P_{s,Tok1}$*	4.6642	1e-18 m/s	Estimated from [59]
α_{Tok1}	231.6		Taken from [38]
β_{Tok1}	8122.7		Taken from [38]
$l_{Tok1,ext}$	0.0		Taken from [38]
$l_{Tok1,int}$	0.76875		Taken from [38]
$k_{Tok1,1}$	3.4*1e+7	s^{-1}	Taken from [38]
$k_{Tok1,2}$	3.4*1e+7	s^{-1}	Taken from [38]

$k_{Tok1,OR}$	1e+4	s^{-1}	Taken from [38]
$k_{Tok1,RO}$	1e+4	s^{-1}	Taken from [38]
$Km_{Tok1,Hog1}$	2*1e-5	mM	
Trk system			
k_{Trk}*	54.0	1e-18 mol/(s*V^2)	Estimated from [6,55]
$Km_{Trk\_high,K}$	0.01	mM	Taken from [39]
$Km_{Trk\_high,Na}$	100.0	mM	
$Km_{Trk\_medium,K}$	0.4	mM	Taken from [39]
$Km_{Trk\_medium,Na}$	40.0	mM	
$Km_{Trk,Ppz}$	1e-5	mM	Estimated from [9,55]
$Km_{Trk,Cn}$	5*1e-4	mM	Estimated from [9,55]
Ppz phosphatases			
Ppz^0	6.607*1e-5	mM	[60]
Km_{Ppz}	7.94*1e-5	mM	
NSC1			
k_{NHS1}*	6*1e-4	1e-18 mol/(s*mM)	
$Km_{NSC1,K}$	60.0	mM	Taken from [61]
$Km_{NSC1,Na}$	100.0	mM	
H^+ production			
K_{H_prod}	5	1e-18 mol/s	
H^+ uptake			
k_{H_uptake}	10.0	1e-18 mol/(s*V)	

Pma1p			
k_{Pma1}*	4.8*1e+4	1e-18 mol/(s*mM^2)	Estimated from [45]

* The values of these parameters were adjusted during the model integration step such that simulation results are consistent with experimental data.
