# Supplementary material for: An Integrative Model of Ion Regulation in Yeast
Source: PLoS Comput Biol. 2013 Jan 17;9(1):e1002879. doi: 10.1371/journal.pcbi.1002879 (PMC3547829; doi:10.1371/journal.pcbi.1002879)
Supplement: Table S3 — Parameters for the signaling module. (PDF) [file pcbi.1002879.s011.pdf]

Table S3. Parameters for the transcriptional module

**Model/** Parameters	**Value**	**Unit**	**Reference**
Hog1 Model			
α_{Hog1}	0.398 *1e+6	$J \cdot m^{-3}$	Taken from [50]
β_{Hog1}	0.2992		Taken from [50]
γ_{Hog1}	0.9626*1e+6	$J \cdot m^{-3}$	Taken from [50]
w_{Hog1}	4.688*1e+3	$J \cdot m^{-3} \cdot mM^{-1}$	Taken from [50]
K_{pho}^{Pbs2}	0.6537	s^{-1}	Taken from [50]
K_{depho}^{Pbs2}	0.224	s^{-1}	Taken from [50]
K_{pho}^{Hog1}	186.67	$mM^{-1} \cdot s^{-1}$	Taken from [50]
$K_{depho}^{Hog1PPc}$	1.5*1e-3	s^{-1}	Taken from [50]
K_{imp}^{Hog1c}	0.0125	s^{-1}	Taken from [50]
K_{exp}^{Hog1n}	0.106	s^{-1}	Taken from [50]
$K_{imp}^{Hog1PPc}$	0.02	s^{-1}	Taken from [50]
$K_{exp}^{Hog1PPn}$	0.1179	s^{-1}	Taken from [50]
$K_{depho}^{Hog1PPn}$	0.0686	s^{-1}	Taken from [50]
K_{s0}^{Glyc}	0.0875	$mM \cdot s^{-1}$	Taken from [50]
K_{s1}^{Glyc}	0.9357	$mM \cdot s^{-1}$	Taken from [50]
K_{s2}^{Glyc}	18.983	s^{-1}	Taken from [50]
K_{exp0}^{Glyc}	8.333*1e-5	s^{-1}	Taken from [50]
K_{exp1}^{Glyc}	4.938*1e-4	s^{-1}	Taken from [50]

K_{s0}^{Yt}	2.018*1e-7	$\mu M \cdot min^{-1}$	Taken from [50]
K_{s1}^{Yt}	0.02	s^{-1}	Taken from [50]
K_{t}^{Yt}	1.489*1e-4	s^{-1}	Taken from [50]
Calcineurin Model			
C_{Ca}	2.5*1e-6	$mM\ s^{-1}$	
d_{Ca}	0.05	s^{-1}	Estimated from [25]
h_Na_cyt*	12		Estimated from [25,27]
h_Na_ext*	2		Estimated from [25,27]
$k_{Ca,cyt}$*	8.0*1e-6	$mM\ s^{-1}$	Estimated from [25,27]
$k_{Ca,ext}$*	2.4*1e-5	$mM\ s^{-1}$	Estimated from [25,27]
Km_{Ca_cyt}	180.0	mM	Estimated from [25,27]
Km_{Ca_ext}	500.0	mM	Estimated from [25,27]
$k_{Ca,pH}$*	1.5*1e-5	$mM\ s^{-1}$	
$k_{CN,a}$	1.0e+9	$mM^{-3}\ s^{-1}$	Estimated from [62]
$k_{CN,da}$	5.0*1e-4	s^{-1}	Estimated from [62]
$k_{CN\_Ppz,da}$*	200.0	$mM^{-1}\ s^{-1}$	Estimated from [63]
C_{Crz1}	7.866*1e-7	$mM\ s^{-1}$	
d_{Crz1}	4.1*1e-3	s^{-1}	
k_{Crz1}*	0.1	s^{-1}	
Nrg1p Model			
C_{Nrg1}	4*1e-7	$mM\ s^{-1}$	
d_{Nrg1}	7.5*1e-4	s^{-1}	Estimated from [64]

$k_{Nrg1,pH}$*	5.25*1e-8	mM s^{-1}	Estimated from [34,65]
ENA1 Model			
$C_{ENA1,Nrg1}$*	1.5*1e-6	mM s^{-1}	Estimated from [33,60,66]
$Km_{ENA1,Nrg1}$*	1.143.0*1e-4	mM	Estimated from [34,65]
h_ENA1Nrg1*	3		
$k_{ENA1,Crz1}$*	4.5*1e-5	s^{-1}	Estimated from [27,33]
$k_{ENA1,Hog1}$*	1.5*1e-6	s^{-1}	Estimated from [27,33]
$d_{ENA1mRNA}$*	0.00683	s^{-1}	Estimated from [66]
kt_{Ena1}	0.0205	s^{-1}	
d_{Ena1}	1.33*1e-3	s^{-1}	Estimated from [64]

* The values of these parameters were adjusted during the model integration step such that simulation results are consistent with experimental data.
