# Supplementary material for: An Integrative Model of Ion Regulation in Yeast
Source: PLoS Comput Biol. 2013 Jan 17;9(1):e1002879. doi: 10.1371/journal.pcbi.1002879 (PMC3547829; doi:10.1371/journal.pcbi.1002879)
Supplement: Table S4 — Basic physiological parameters. (PDF) [file pcbi.1002879.s012.pdf]

Table S4. Basic physiological parameters

**Parameter**	**Description**	**Value**	**Unit**	**Reference***
R	Gas constant	8.31447		
T	Absolute temperature	310	K	
F	Faraday constant	96485	C/mol	
G_{0_ATP}	Gibbs energy for ATP hydrolysis	-36.03*1e+3	J	
P_K	Membrane permeability to potassium ion	1e-6	μm/s	[67,68]
P_{Na}	Membrane permeability to sodium ion	3.8*1e-8	μm/s	[67,68]
Cm	Membrane capacitance	1e-2	farad/m^2	[2]
D_{cell}	Cell diameter	4	μm	[69]
$Volume_{Cell}$	Cell volume	33.510	$μm^3$	
S_{mem}	Cell surface area	50.2655	$μm^2$	
Lp	Hydraulic membrane permeability	1.19*1e+6	m^4/(J*s)	[51]
G_{EK}	Geometrical factor	7.85*1e-11	m^2	[51]
$Osmo_{cyt}^0$	Total concentration of initial intracellular osmolytes	600	mM	[51]

$Osmo_{ext}^{0}$	Total concentration of initial extracellular osmolytes	250	mM	[51]
Anion	Intracellular negative charges that balances H^+, K^+ and Na^+	300	mM	
$Turgor^0$	Initial turgor pressure	0.875*1e+6	J/m^3	[51]
r_{vol}	Volumetric elastic modulus	0.63		[51]
[ATP]	ATP concentration	2.6	mM^{-1}	[70,71]
[ADP]	ADP concentration	1.0	mM^{-1}	[70,71]
[Pi]	Phosphate concentration	3.0	mM^{-1}	[70,71]
