# Supplementary material for: An Integrative Model of Ion Regulation in Yeast
Source: PLoS Comput Biol. 2013 Jan 17;9(1):e1002879. doi: 10.1371/journal.pcbi.1002879 (PMC3547829; doi:10.1371/journal.pcbi.1002879)
Supplement: Table S5 — Initial concentrations for the ODE system. (PDF) [file pcbi.1002879.s013.pdf]

Table S5. Initial concentrations for the ODE system

Parameter	Description	Value	Unit
Init_H	Initial intracellular H^+ concentration	1e-4 (pH7)	mM
Init_K	Initial intracellular K^+ concentration	200	mM
Init_Na	Initial intracellular Na^+ concentration	100	mM
Init_CytVolume	Initial volume for the osmotically changeable compartment	20.1062	μm^3
Init_Pbs2	Initial unphosphorylated Pbs2 concentration	1.231*1e-4	mM
Init_Pbs2PP	Initial phosphorylated Pbs2 concentration	6.16*1e-7	mM
Init_Hog1c	Initial unphosphorylated cytosolic Hog1p concentration	3.426*1e-4	mM
Init_Hog1PPc	Initial phosphorylated cytosolic Hog1p concentration	4.443*1e-6	mM
Init_Hog1n	Initial unphosphorylated nuclear Hog1p concentration	2.918*1e-4	mM
Init_Hog1PPn	Initial phosphorylated nuclear Hog1p concentration	3.38*1e-6	mM
Init_Glycerol	Initial intracellular glycerol concentration	576	mM
Init_Yt	Initial glycerol-producing protein concentration	1.811*1e-3	mM
Init_z1	Initial concentration of glycerol metabolism enzyme 1	3.38*1e-6	mM

Init_z2	Initial concentration of glycerol metabolism enzyme 2	3.38*1e-6	mM
Init_z3	Initial concentration of glycerol metabolism enzyme 3	3.38*1e-6	mM
Init_z4	Initial concentration of glycerol metabolism enzyme 4	3.38*1e-6	mM
Init_Ca	Initial Ca^{2+} concentration	5*1e-5	mM
Init_CN_{off}	Initial inactive calcineurin concentration	1.1628*1e-3	mM
Init_CN_{on}	Initial active calcineurin concentration	0	mM
Init_Crz1	Initial Crz1p concentration	1.916*1e-4	mM
Init_Nrg1	Initial Nrg1p protein concentration	9.1838*1e-5	mM
Init_Ena1mRNA	Initial *ENA1* mRNA concentration	7*1e-6	mM
Init_Ena1	Initial Ena1p protein concentration	1.1364*1e-4	mM

*Note: All the results in this study were obtained by firstly, simulating the ODEs for 60,000 seconds (-60,000<t<0) initial conditions presented in this table allowing the system to reach a steady state, and then, imposing stress conditions in each experiment (t>0). The parameters were chosen such that the steady state value of each protein concentration in unstressed growth condition is similar to the measurement in Ref. [60], i.e. the initial protein concentrations in this table.
