# Supplementary material for: An Integrative Model of Ion Regulation in Yeast
Source: PLoS Comput Biol. 2013 Jan 17;9(1):e1002879. doi: 10.1371/journal.pcbi.1002879 (PMC3547829; doi:10.1371/journal.pcbi.1002879)
Supplement: Table S6 — Parameter sensitivity to experimental data using Latin-Hypercube Sampling. (PDF) [file pcbi.1002879.s014.pdf]

Table S6. Parameter sensitivity to experimental data using Latin-Hypercube Sampling. Parameter values that are significantly different from 0, i.e. parameters that has a significantly strong impact on the difference between simulation and data, are shown in shaded numbers ($p<0.001$).

**Model/** Parameters	**Partial rank correlation coefficient**		
	**Fig. 2C**	**Fig. 3B**	**Fig. 3C**
Transporters			
k_{Ena1}	0.0756	0.0430	-0.1441
k_{Nha1}	0.0141	0.0033	0.0370
$P_{s,Tok1}$	0.0790	0.0194	0.1114
k_{Trk}	0.1696	0.1902	0.0826
k_{Pma1}	0.0018	0.0019	0.0066
Hog1 Model			
$_{\mathrm{Hog1}}$	0.0617	0.0326	0.0582
$_{\mathrm{Hog1}}$	0.0329	0.0238	0.0006
$_{\mathrm{Hog1}}$	-0.0701	-0.0271	-0.0268
w_{Hog1}	-0.0071	-0.0055	-0.0049
K_{pho}^{Pbs2}	-0.0038	-0.0135	-0.0004
K_{depho}^{Pbs2}	-0.0180	-0.0136	-0.0086
K_{pho}^{Hog1}	0.0149	-0.0031	0.0228

$K_{depho}^{Hog1PPc}$	-0.0048	0.0030	0.0008
K_{imp}^{Hog1c}	0.0075	0.0018	0.0077
K_{exp}^{Hog1n}	0.0077	0.0028	0.0089
$K_{imp}^{Hog1PPc}$	-0.0041	0.0157	0.0116
$K_{exp}^{Hog1PPn}$	0.0030	-0.0143	0.0015
$K_{depho}^{Hog1PPn}$	-0.0208	-0.0168	-0.0185
K_{s0}^{Glyc}	0.0222	0.0123	0.0240
K_{s1}^{Glyc}	-0.0237	-0.0186	-0.0158
K_{s2}^{Glyc}	-0.0145	-0.0230	0.0126
K_{exp0}^{Glyc}	-0.0235	-0.0145	-0.0751
K_{exp1}^{Glyc}	0.0150	-0.0007	0.0114
K_{s0}^{Yt}	0.0062	0.0037	0.0061
K_{s1}^{Yt}	-0.0131	-0.0162	0.0074
K_{t}^{Yt}	0.0049	0.0044	-0.0134
Calcineurin Model			
C_{Ca}	-0.0047	0.0026	0.0345
d_{Ca}	-0.0094	-0.0241	-0.0580
$k_{Ca,cyt}$	0.0007	-0.0056	0.0040
$k_{Ca,ext}$	0.0352	0.0575	0.0461
Km_{Ca_cyt}	0.0186	0.0317	0.0132
Km_{Ca_ext}	0.0007	-0.0605	-0.0504
$k_{Ca,pH}$	0.0047	-0.0080	0.0037

$k_{CN,a}$	0.0009	0.0025	0.0236
$k_{CN,da}$	-0.0063	-0.0022	-0.0134
$k_{CN\_Ppz,da}$	-0.0609	-0.0743	-0.0801
C_{Crz1}	0.0034	0.0052	-0.0167
d_{Crz1}	-0.0420	-0.0638	-0.1092
k_{Crz1}	0.0418	0.0610	0.1376
Nrg1p Model			
C_{Nrg1}	-0.0241	-0.0277	0.0107
d_{Nrg1}	0.0228	0.0417	-0.0028
$k_{Nrg1,pH}$	0.0105	0.0120	0.0018
ENA1 Model			
$C_{ENA1,Nrg1}$	-0.0055	-0.0062	-0.0282
$Km_{ENA1,Nrg1}$	-0.0349	-0.0406	0.0034
$k_{ENA1,Crz1}$	0.0045	0.0255	0.0667
$k_{ENA1,Hog1}$	0.0430	0.0436	0.0193
$d_{ENA1mRNA}$	-0.0554	-0.0135	-0.0931
kt_{Ena1}	0.0678	0.0905	0.0984
d_{Ena1}	-0.0663	-0.0823	-0.0080
