# Supplementary material for: An Integrative Model of Ion Regulation in Yeast
Source: PLoS Comput Biol. 2013 Jan 17;9(1):e1002879. doi: 10.1371/journal.pcbi.1002879 (PMC3547829; doi:10.1371/journal.pcbi.1002879)
Supplement: Text S1 — Supplementary material. (PDF) [file pcbi.1002879.s015.pdf]

Supporting information (Text S1) for
'An integrative model of ion regulation in yeast'

Ruian Ke[1,2*†], Piers J. Ingram[1,2], Ken Haynes[3†]

[1]Department of Mathematics, [2]Centre for Integrative Systems Biology (CISBIC), and [3]Department of Microbiology, Imperial College London, London, SW7 2AZ, UK

[†]Present address:

R.K.: Department of Ecology and Evolutionary Biology, University of California, Los Angeles, 610 Charles E. Young Dr. South, Los Angeles, CA 90095

K. H.: Biosciences, College of Life & Environmental Science, Geofrey Pope Building, Stocker Road, Exeter, EX4 4AD, United Kingdom

[*]To whom correspondence should be addressed:

Email: Ruian Ke (ruian@ucla.edu)

Table of contents

1 Overview **3**

2 Modeling the transporters **6**

2.1 Membrane potential 7

2.2 Na^+ and K^+ transporters 8

2.3 H^+ production, import and export 23

2.4 Membrane leakage 26

2.5 ODEs for ion transport 27

3 Modeling transcriptional regulation **28**

3.1 The HOG pathway 29

3.2 The calcineurin pathway 32

3.3 Transcription regulation of Nrg1p 37

3.4 *ENA1* gene expression 38

4 Modeling volume change **39**

5 Modeling mutants **41**

Supplementary references **43**

1 Overview

Below we present an overview of the biology of the regulatory components in response to NaCl, osmotic, KCl and alkaline pH stress conditions that are considered in this study (also see Fig. 1 and Table 1 in the main text).

Transporters

Saccharomyces cerevisiae cells create a large gradient of protons across the plasma membrane. This proton gradient is used as an energy source by the cell to transport cations, amino acids, phosphates and other small nutrient molecules across the cell membrane [1]. Potassium ions (K^+) are actively taken up by K^+ transporters to balance the charge difference generated by the proton extrusion. Due to similar chemical properties with K^+, sodium ions (Na^+) are able to enter the cell through the same transporters as K^+. Since a high intracellular Na^+ concentration is toxic for the cell and Na^+ is the most abundant cation in most natural environments, the cell has evolved complex transport mechanisms to tightly regulate intracellular K^+ and Na^+ levels by actively taking in K^+ and pumping out Na^+ [2]. Under stress conditions, such as saline stress, osmotic stress and alkaline pH stress, yeast cells maintain intracellular caion homeostasis by both post-translational modification of the activities and transcriptional

regulation of gene expression of various transporters. In this study, we mainly focus on the activity and regulation of 6 major transporters/transporter system that regulate intracellular H^+, K^+ and Na^+ concentrations: Pma1p, the Trk system (including Trk1,2p), Ena1p, Nha1p, Tok1p and the as yet genetically uncharacterized non-selective cation channel NSC1.

The proton gradient is created through the activity of the H^+-ATPase, Pma1p. It actively extrudes protons using energy released from ATP hydrolysis [3]. K^+ uptake is primarily mediated by the Trk system [4,5,6]. It has two states depending on its affinity to K^+: the high affinity state and the medium affinity state. In high Na^+ environments, Trk1p switches to the high affinity state to discriminate K^+ when Na^+ becomes excessive [7,8]. The low-affinity potassium uptake is mediated at least in part by the non-specific cation transporter, NSC1 [9]. The outward-rectifying channel Tok1p extrudes intracellular K^+ when K^+ becomes excessive [10,11]. The Na^+,K^+/H^+ antiporter, Nha1p [12,13] and the Na^+-ATPase, Ena1p, pump out intracellular Na^+ [14,15]. Under neutral conditions, excessive intracellular Na^+ is extruded primarily through Nha1p. Under high salt conditions and high pH conditions, the expression of *ENA1* gene is up-regulated and Ena1p becomes the primary Na^+ pump [12].

Post-translational regulation

The activities of these transporters are under complex regulation, and are highly dependent on the environment. The post-translational modulation of the transporters in response to high salt, high osmolarity and high alkaline pH stresses are mainly mediated by the protein kinase Hog1p [16], calcineurin [17] and the protein phosphatases Ppz1,2p [18,19]. Hog1p is a protein kinase that is activated in response to hyper-osmotic stress [16]. Hog1p modifies both the activity and gene expressions of those enzymes involved in glycerol synthesis [20,21,22]. In addition, Hog1p interacts with Nha1p and Tok1p at the plasma membrane to decrease Na^+ toxicity upon activation [23]. In yeast cells, both osmotic stress and alkaline pH stress induce a rapid accumulation of Ca^{2+} ions in the cytosol, which in turn activate calcineurin [24,25]. Calcineurin plays a number of important roles in the adaptation to NaCl and high pH stresses, and deletion of calcineurin subunits, or addition of the calcineurin inhibitor FK506, results in growth sensitivity under those conditions [26,27]. The Ser/Thr protein phosphatases, Ppz1,2p, are involved in many cellular processes, including salt tolerance [19,28,29,30]. They negatively regulate calcineurin activity and switch the Trk system from high affinity to low affinity [18,31]. It has been shown that the activities of Ppz proteins are dependent on intracellular pH [31,32]. This is a result of the pH dependent binding between Ppz proteins with a Ppz inhibitor protein, Hal3p.

Transcriptional regulation

The transcriptional responses to osmotic stress, salt stress and alkaline pH stress involve several signaling pathways. The calcineurin pathway is involved in adaptation to all three stress conditions, though not with equal importance. This pathway is not required under sorbitol or KCl stress, however, in high Na^+ or high pH conditions, its activation is essential for growth [17,26,33]. Hyper-osmotic stress adaptation is critically dependent on Hog1p activation through the HOG pathway. Several pathways are involved in the transcriptional response to alkaline pH. In terms of ion homeostasis, one of the important responses is the repression of *NRG1*, encoding the transcription factor Nrg1p [34]. Under all three stress conditions mentioned, *ENA1* gene expression is highly up-regulated, highlighting the importance of this Na^+ ATPase in ion homeostasis during stress adaptation.

2 Modeling the transporters

In this section, the sub-models describing transporters and post-translational modifications to these transporters are presented in detail. Since transporter activity is generally dependent on the plasma membrane potential, we first describe the equation for

calculating the plasma membrane potential in Section 2.1. The equations for the Na^+ and K^+ transporters are explained in Section 2.2, followed by the sub-models for the H^+ transport in Section 2.3 and the model describing the membrane leakage of K^+ and Na^+ in Section 2.4. Finally, the ODE describing the temporal changes of intracellular K^+, Na^+ and H^+ are shown in Section 2.5. The values of model parameters and references from which the parameter values are determined in this section are listed in Table S2 and S3.

2.1 Membrane potential

The membrane potential (*Em*) is calculated directly from the net charge inside the cell by adding up the number of major mono-valent ions, i.e. H^+, K^+, Na^+, and the negatively changed anions. The equation is given by

$$Em(t) = \left(H^+_{cyt}(t) + K^+_{cyt}(t) + Na^+_{cyt}(t) - Anion_{cyt}\right) \cdot \frac{F}{Cm \cdot S_{Mem}} \quad (2.1),$$

where ${H_{cyt}}^+$, ${K_{cyt}}^+$ and ${Na_{cyt}}^+$ are the amounts of cytosolic H^+, K^+ and Na^+ molecules, respectively, and their levels are dependent on transporter activities. $Anion_{cyt}$ is the concentration of anions in the cell that balance positive charges of H^+, K^+ and Na^+. We assume $Anion_{cyt}$ is constant. F is the Faraday constant, Cm is the membrane capacitance and S_{Mem} is the surface area of the cell membrane. The values of these constants can be found in Table S4.

2.2 Na^+ and K^+ transporters

Both Na^+ and K^+ are able to bind to the same site on many transporters, e.g. Nha1p, Ena1p and the Trk1,2p transporters, albeit with different affinities. We first derive equations for the probability of competitive binding at a K^+/Na^+ binding site of a transporter in Section 2.2.1, and apply this general result to transporters that transport both Na^+ and K^+ in following sections.

2.2.1 The Na^+ and K^+ Competitive Binding Probability (CBP)

The chemical equation for the binding and unbinding of Na^+ and K^+ to the transporters is:

$$[Na^+]+[T_{free}]\leftrightarrow[T_{Na}],\quad [K^+]+[T_{free}]\leftrightarrow[T_K],$$

where T_{free} is the unbound transporter, T_{Na} is the Na^+ bound transporter, and T_k is the K^+ bound transporter. Then, the total amount of this transporter (T^0) is

$$T^0=T_{free}+T_{Na}+T_K.$$

If we assume the two reactions are in equilibrium and solve the resulting equilibrium mass-action equations, we obtain the probabilities that the transporter is in the unbound state (P_{free}), in the Na^+ bound state (P_{Na}) and in the K^+ bound state (P_K):

$$\mathrm{P}_{free}([K^+],[Na^+])=T_{free}([K^+],[Na^+])/T^0=\frac{1}{1+[K^+]/Km_{T,K}+[Na^+]/Km_{T,Na}} \quad (2.2),$$

$$\mathrm{P}_{Na}([K^+],[Na^+]) = T_{Na}([K^+],[Na^+])/T^0 = \frac{[Na^+]/Km_{T,Na}}{1+[K^+]/Km_{T,K} + [Na^+]/Km_{T,Na}}$$

(2.3),

$$\mathrm{P}_{K}([K^+],[Na^+]) = T_{K}([K^+],[Na^+])/T^0 = \frac{[K^+]/Km_{T,K}}{1+[K^+]/Km_{T,K} + [Na^+]/Km_{T,Na}}$$

(2.4),

where $Km_{T,Na}$, $Km_{T,K}$ are the dissociation constants for the binding of the transporter to Na^+ and K^+, respectively. We refer to these three equations as the Competitive Binding Probability (CBP) equations below.

2.2.2 Ena1p

Ena1p catalyzes ATP hydrolysis to extrude Na^+ and K^+. The binding probabilities of Ena1p to Na^+ and K^+ can be derived from equations (2.3 and 2.4) above:

$$\mathrm{P}_{Ena1,Na} = \frac{[Na^+]_{cyt}/Km_{Ena1,Na}}{1+[K^+]_{cyt}/Km_{Ena1,K} + [Na^+]_{cyt}/Km_{Ena1,Na}} \quad (2.5),$$

$$\mathrm{P}_{Ena1,K} = \frac{[K^+]_{cyt}/Km_{Ena1,K}}{1+[K^+]_{cyt}/Km_{Ena1,K} + [Na^+]_{cyt}/Km_{Ena1,Na}} \quad (2.6),$$

where $Km_{Ena1,Na}$ and $Km_{Ena1,K}$ are dissociation constants for the binding affinities to Na^+ and K^+, respectively.

The chemical reaction for Ena1p transport activity is

$$[Na^+/K^+]_{cyt} + [ATP] + [Ena1] \leftrightarrow [Na^+/K^+]_{ext} + [ADP] + [Pi] + [Ena1].$$

Then, the equations describing Na^+ and K^+ fluxes through Ena1p under unstressed conditions ($J^0_{Ena1,Na}$ and $J^0_{Ena1,K}$, respectively) can be derived as following [35]:

$$J^0_{Ena1,Na} = k_{Ena1} \cdot ([Na^+]_{cyt} \cdot [ATP] - [Na^+]_{ext} \cdot [ADP] \cdot [Pi] \cdot \exp(\frac{G_{0\_ATP} - F \cdot Em}{R \cdot T}))$$

$$J^0_{Ena1,K} = k_{Ena1} \cdot ([K^+]_{cyt} \cdot [ATP] - [K^+]_{ext} \cdot [ADP] \cdot [Pi] \cdot \exp(\frac{G_{0\_ATP} - F \cdot Em}{R \cdot T}))$$

where k_{Ena1} is a flux constant.

To account for the both the binding probabilities to Na^+ and K^+ and changes in the number of Ena1p molecules on the plasma membrane due to the regulation of Ena1p in response to stressful conditions, we model the total fluxes of Na^+ and K^+ through Ena1p ($J_{Ena1,Na}$ and $J_{Ena1,K}$, respectively) as the following:

$$\begin{aligned} J_{Ena1,Na} &= \mathrm{P}_{Ena1,Na} \cdot \frac{Ena1(t)}{Ena1^0} \cdot J^0_{Ena1,Na} \\ &= \mathrm{P}_{Ena1,Na} \cdot \frac{Ena1(t)}{Ena1^0} \cdot k_{Ena1} \cdot ([Na^+]_{cyt} \cdot [ATP] - [Na^+]_{ext} \cdot [ADP] \cdot [Pi] \cdot \exp(\frac{G_{0\_ATP} - F \cdot Em}{R \cdot T})) \end{aligned}$$

(2.7),

$$\begin{aligned} J_{Ena1,K} &= \mathrm{P}_{Ena1,K} \cdot \frac{Ena1(t)}{Ena1^0} \cdot J^0_{Ena1,K} \\ &= \mathrm{P}_{Ena1,K} \cdot \frac{Ena1(t)}{Ena1^0} \cdot k_{Ena1} \cdot ([Na^+]_{cyt} \cdot [ATP] - [Na^+]_{ext} \cdot [ADP] \cdot [Pi] \cdot \exp(\frac{G_{0\_ATP} - F \cdot Em}{R \cdot T})) \end{aligned}$$

(2.8),

where $Ena1^0$ is the amount of Ena1p in unstressed conditions, and $Ena1(t)$ the total amount of Ena1p during the simulation. The ratio of $Ena1(t)$ over $Ena1^0$ describes the relative changes in the amount of Ena1p. The ODE describing $Ena1(t)$ under different

stress conditions is given in Section 3.4. G_{0_ATP} is the free energy released by ATP hydrolysis, R is the gas constant, T is the absolute temperature, F is the Faraday constant and Em is the membrane potential.

Note that, since there is no strong evidence showing that ATP levels are altered notably within the time scale of stress conditions we consider in this study, we assume that the values of *[ADP][Pi]], and [ATP]* are constants (see Table S4).

2.2.3 Nha1p

Nha1p is a plasma membrane localized $H^+/K^+,Na^+$ electrogenic antiporter, i.e. transporting more H^+ than K^+/Na^+ ions [36]. Under high Na^+ stress, Nha1p is phosphorylated by Hog1p, and this phosphorylation is important for extrusion of excessive intracellular Na^+ [23]. Later experiments demonstrated that the potassium efflux through Nha1p is inhibited in a Hog1p dependent manner under osmotic stress [37]. These data suggest that the phosphorylation of Nha1p by Hog1p increases its maximal activity and decreases the Nha1p affinity to K^+. In this study, we assume that Nha1p exists in two states, the un-phosphorylated state (low activity state) and phosphorylated state (high activity state). Phosphorylation by Hog1p increases the activity of Nha1p and decreases its affinity to K^+.

In the following, we firstly derive the probabilities of Nha1p in the two states and the probabilities of its binding to Na^+ and K^+, respectively, and then, we present the equations for the Na^+ and K^+ fluxes through Nha1p.

Since Hog1p modifies Nha1p post-transcriptionally, we assume this reaction is very quick and the levels of Nha1p in the phosphorylated and unphosphorylated states are at quasi-equilibrium. Then, the probabilities that Nha1p is in the phosphorylated state (P_{Nha1_low}, low affinity to K^+) and the un-phosphorylated state (P_{Nha1_high}) can be approximated as:

$$P_{Nha1\_high} = \frac{Km_{Nha1,Hog1}}{Km_{Nha1,Hog1} + Hog1PP_{cyt}(t)} \qquad (2.9),$$

$$P_{Nha1\_low} = \frac{Hog1PP_{cyt}(t)}{Km_{Nha1,Hog1} + Hog1PP_{cyt}(t)} \qquad (2.10),$$

where $Km_{Nha1,Hog1}$ is the dissociation constant for Hog1p regulation, and $Hog1PP_{cyt}(t)$ is the time dependent level of phosphorylated Hog1p in the cytosol. The equations describing $Hog1PP_{cyt}(t)$ is given in Section 3.1.

We consider the binding of Nha1p to Na^+ and K^+ separately for the phosphorylated and the unphosphorylated Nha1p. From the CBP equations, the binding probabilities to Na^+ and K^+ for the un-phosphorylated Nha1p (high affinity to K^+) can be derived:

$$P_{Nha1\_high,Na}([K^+]_{cyt},[Na^+]_{cyt}) = \frac{[Na^+]_{cyt} / Km_{Nha1,Na}}{1+[K^+]_{cyt} / Km_{Nha1\_high,K} + [Na^+]_{cyt} / Km_{Nha1,Na}} \quad (2.11),$$

$$P_{Nha1\_high,K}([K^+]_{cyt},[Na^+]_{cyt}) = \frac{[K^+]_{cyt} / Km_{Nha1\_high,K}}{1+[K^+]_{cyt} / Km_{Nha1\_high,K} + [Na^+]_{cyt} / Km_{Nha1,Na}} \quad (2.12),$$

where $Km_{Nha1,Na}$ is the dissociation constant of Nha1p to Na^+ and $Km_{Nha1\_high,K}$ is the dissociation constant of un-phosphorylated Nha1p to K^+.

Similarly, for the phosphorylated Nha1p (lower affinity to K^+), the binding probabilities to K^+ and Na^+ are:

$$P_{Nha1\_low,Na}([K^+]_{cyt},[Na^+]_{cyt}) = \frac{[Na^+]_{cyt} / Km_{Nha1,Na}}{1+[K^+]_{cyt} / Km_{Nha1\_low,K} + [Na^+]_{cyt} / Km_{Nha1,Na}} \quad (2.13),$$

$$P_{Nha1\_low,K}([K^+]_{cyt},[Na^+]_{cyt}) = \frac{[K^+]_{cyt} / Km_{Nha1\_low,K}}{1+[K^+]_{cyt} / Km_{Nha1\_low,K} + [Na^+]_{cyt} / Km_{Nha1,Na}} \quad (2.14),$$

where $Km_{Nha1\_low,K}$ is the dissociation constant of the phosphorylated Nha1p to K^+.

The chemical reaction for Nha1p is:

$$3\cdot[H^+]_{ext} + 2\cdot[K^+/Na^+]_{cyt} + [Nha1p] \leftrightarrow 3\cdot[H^+]_{cyt} + 2\cdot[K^+/Na^+]_{ext} + [Nha1p]$$

The equation describing the flux can be written as the following form:

$$J_{Nha1,K} = V_{Nha1,K}\cdot([H^+]^3_{ext}\cdot[K^+]^2_{cyt} - [H^+]^3_{cyt}\cdot[K^+]^2_{ext}\cdot\exp(\frac{G_{0\_ATP} - F\cdot Em}{R\cdot T})),$$

$$J_{Nha1,Na} = V_{Nha1,Na}\cdot([H^+]^3_{ext}\cdot[Na^+]^2_{cyt} - [H^+]^3_{cyt}\cdot[Na^+]^2_{ext}\cdot\exp(\frac{G_{0\_ATP} - F\cdot Em}{R\cdot T}))$$

(2.15) ,

where $V_{Nha1,K}$ and $V_{Nha1,Na}$ describes the dependence of the rate of fluxes on the state of Nha1p and the binding probabilities of Nha1p, the expressions for $V_{Nha1,K}$ and $V_{Nha1,Na}$ are :

$$V_{Nha1,K} = k_{Nha1\_low} \cdot P_{Nha1\_low} \cdot P_{Nha1\_low,K} + k_{Nha1\_high} \cdot P_{Nha1\_high} \cdot P_{Nha1\_high,K}, \quad (2.16)$$

and

$$V_{Nha1,Na} = k_{Nha1\_low} \cdot P_{Nha1\_low} \cdot P_{Nha1\_low,Na} + k_{Nha1\_high} \cdot P_{Nha1\_high} \cdot P_{Nha1\_high,Na} \quad (2.17),$$

where k_{Nha1_low} and k_{Nha1_high} are the rate constant for Nha1p at phosphorylated and unphosphorylated states, respectively. The first terms on the right hand side of Eqn 2.16 and 2.17 describe the dependence of K^+ and Na^+ fluxes through phosphorylated Nha1p, respectively, and the second terms describe the dependence of the fluxes through unphosphorylated Nha1p.

2.2.4 Tok1p

Tok1p is an outward rectifying K^+ channel at the plasma membrane. Under NaCl stress, phosphorylated Hog1p interacts with Tok1p at the plasma membrane, and this interaction is shown to be important to cellular adaptation to NaCl stress [23].

Loukin and Saimi proposed a biophysical model that explains the gating properties of Tok1p very well [38]. Most of the model results are consistent with experimental

measurements, however, the predictions from their model have to be corrected using the Goldman-Hodgkin-Katz (GHK) equation if the intracellular and extracellular K^+ concentrations are not symmetric, which is problematic for our study. Therefore, we made modifications to their model and the modified model is able to produce results that are consistent with the published measurements for non-symmetric K^+ concentration conditions. A brief explanation of the Loukin and Saimi model and the modifications is given below.

Loukin and Saimi Model and modifications

Based on the measurements by Lesage and colleagues [10], Loukin and Saimi assumed four different states for Tok1p, R_I, R_{IO}, R_O and O, where R_I, R_{IO}, R_O are sub-states of the blocked state and O is the open state. The probability that Tok1p is in the open (O) state is given by the following:

$$P_{Tok1,O} = \frac{[O]}{[O]+[R_I]+[R_{IO}]+[R_O]} \qquad (2.18),$$

where the possible transitions between all of the four states are: $R_O \leftrightarrow R_{IO} \leftrightarrow R_I \leftrightarrow O$.

The rates of these transitions are governed by membrane potential and intra- and extra-cellular K^+ concentration. If we assume the reactions are in equilibrium, the

relative distributions of the four states are given by the following equations:

$$O = \frac{k_{Tok1,RO} \cdot R_I}{k_{Tok1,OR}}$$

$$R_I = \frac{k_{Tok1,OR} \cdot O + k'_{Tok1,-1} \cdot R_{IO}}{k_{Tok1,RO} + k'_{Tok1,1} \cdot [K^+]_{ext}}$$

$$R_{IO} = \frac{k'_{Tok1,1} \cdot [K^+]_{ext} \cdot R_I + k'_{Tok1,-2} \cdot [K^+]_{\text{int}} \cdot R_O}{k'_{Tok1,-1} + k'_{Tok1,2}}$$

$$R_O = \frac{k'_{Tok1,2} \cdot R_{IO}}{k'_{Tok1,-2} \cdot [K^+]_{\text{int}}},$$

whereas $k_{Tok1,RO}$ and $k_{Tok1,OR}$ are parameter constants, and the other four parameters are given by the following expressions:

$$k'_{Tok1,1} = k_{Tok1,1} \cdot \exp(-l_{Tok1,ext} \cdot Em/(R \cdot T))$$

$$k'_{Tok1,-1} = \alpha_{Tok1} \cdot k_{Tok1,1} \cdot \exp(l_{Tok1,ext} \cdot Em/(R \cdot T))$$

$$k'_{Tok1,2} = \beta_{Tok1} \cdot k_{Tok1,2} \cdot \exp(-l_{Tok1,\text{int}} \cdot Em/(R \cdot T))$$

$$k'_{Tok1,-2} = k_{Tok1,2} \cdot \exp(l_{Tok1,\text{int}} \cdot Em/(R \cdot T)).$$

The values of $k_{Tok1,RO}$, $k_{Tok1,OR}$, $k_{Tok1,1}$ and $k_{Tok1,2}$ are given in Table 2.

In the Loukin and Saimi model [38], the current through Tok1p is calculated from the conductance of the open state of this transporter and the overall conductance is assumed proportional to the amount of Tok1p in the open state (given by $P_{Tok1,O}$ above). Here, however, we model the current through the open state directly instead, using the Goldman-Hodgkin-Katz (GHK) equation:

$$I_{Tok1} = Ps_{Tok1} \cdot \frac{F^2}{R \cdot T} \cdot Em \cdot \frac{[K^+]_{cyt} - \exp(-\frac{Em \cdot F}{R \cdot T}) \cdot [K^+]_{ext}}{1 - \exp(-\frac{Em \cdot F}{R \cdot T})} \qquad (2.19),$$

where Ps_{Tok1} is the total permeability of Tok1p at the plasma membrane to K^+.

Then, the K^+ flux through Tok1p in the absence of Hog1p interaction is:

$$J'_{Tok1} = P_{Tok1,O} \cdot I_{Tok1} / F,$$

where F is the Faraday constant.

To understand the interaction between Tok1p and Hog1p, we varied Tok1p activity in NaCl stress conditions (see Section 5) and found that increasing Tok1p activity results in higher intracellular Na^+ concentration, whereas, decreasing Tok1p activity results in lower Na^+ concentration (Fig. 3A in the main text). Therefore, we find that Hog1p negatively regulates Tok1p activity through phosphorylation, which is in agreement with previous measurement that K^+ efflux rate is minimal during osmotic stresses [37].

Hence, by taking account of the regulation of Hog1p, the overall Tok1p open probability is modeled as:

$$\begin{aligned} J_{Tok1} &= \frac{Km_{Tok1,Hog1}}{Hog1PP_{cyt}(t) + Km_{Tok1,Hog1}} \cdot J'_{Tok1} \\ &= \frac{Km_{Tok1,Hog1}}{Hog1PP_{cyt}(t) + Km_{Tok1,Hog1}} \cdot P_{Tok1,O} \cdot I_{Tok1} / F \end{aligned} \qquad (2.20),$$

where $Km_{Tok1,Hog1}$ are Michaelis-Menten constants for Hog1p regulation.

2.2.5 The Trk1,2p transport system

The Trk1,2p system exists in two states, a medium affinity state and a high affinity state [39]. The affinity of the Trk1,2p system to K^+ is in the micromolar range when it is in the high affinity state, and in the millimolar range when it is in the medium affinity state (Gaber, Styles et al. 1988; Ramos, Alijo et al. 1994). The transition between the high affinity state and the medium affinity state is mainly mediated by calcineurin and Ppz proteins (Ppz1,2p) [9,33]. It has been shown that activation of calcineurin promotes the Trk1,2p transition from medium affinity to high affinity [33]. Activation of the calcineurin pathway increases the amount of Trk1p at the plasma membrane by up-regulating the expression of *HAL5*, which encodes Hal5p regulatory protein that stabilizes Trk1p at the plasma membrane [40]. The interaction between Ppz proteins and Trk1p converts Trk1p from high affinity to medium affinity [18,31]. In the following, we firstly model the level of Ppz1p, and then describe the model for the Trk1,2p transport system.

Ppz regulatory proteins

The activities of Ppz proteins have been shown to be dependent on intracellular pH [31].

Increase in intracellular pH increases the activity of Ppz. Here, we model the activity of Ppz proteins (*[Ppz]*) as following:

$$[Ppz]=\frac{Km_{Ppz}}{Km_{Ppz}+[H^{+}]_{cyt}}\cdot[Ppz^{0}] \qquad (2.21)\ ,$$

where [Ppz^0] is the total concentration of Ppz protein, and Km_{ppz} is a Michaelis-Menten constant.

Trk transporter system transporter activity

To model the Trk1,2p transporter (the Trk system) activity, we firstly calculate the fractions of the Trk system in the medium affinity state and the high affinity state. Since Ppz proteins negatively regulate the Trk system and calcineurin positively regulates it [18,33], we model the dependence of the probabilities of the Trk system in the medium and high states (P_{Trk_medium}, P_{Trk_high}) as following:

$$P_{Trk\_medium}([Ppz],[CN])=\frac{[Ppz]/Km_{Trk,Ppz}}{[Ppz]/Km_{Trk,Ppz}+[CN]/Km_{Trk,CN}+1} \qquad (2.22)\ ,$$

$$P_{Trk\_high}([Ppz],[CN])=\frac{[CN]/Km_{Trk,CN}+1}{[Ppz]/Km_{Trk,Ppz}+[CN]/Km_{Trk,CN}+1} \qquad (2.23)\ ,$$

where $Km_{Trk,Ppz}$, $Km_{Trk,CN}$ are the dissociation constants for Ppz phosphatase and calcineurin regulations on the Trk system, respectively.

Secondly, we derive the binding probabilities of the Trk system to K^+ and Na^+. From the CBP equations, we have the following equations describing the probabilities that the Trk

system binds to Na^+ and K^+ at high affinity state ($P_{Trk\_high,Na}$ and $P_{Trk\_high,K}$, respectively):

$$P_{Trk\_high,Na}([K^+]_{ext},[Na^+]_{ext}) = \frac{[Na^+]_{ext} / Km_{Trk\_high,Na}}{1+[K^+]_{ext} / Km_{Trk\_high,K} + [Na^+]_{ext} / Km_{Trk\_high,Na}}$$

(2.24) ,

$$P_{Trk\_high,K}([K^+]_{ext},[Na^+]_{ext}) = \frac{[K^+]_{ext} / Km_{Trk\_high,K}}{1+[K^+]_{ext} / Km_{Trk\_high,K} + [Na^+]_{ext} / Km_{Trk\_high,Na}}$$

(2.25) ,

where $Km_{Trk\_high,K}$ and $Km_{Trk\_high,Na}$ are the dissociation constants for the high affinity state of the Trk system for K^+ and Na^+, respectively.

Similarly, the binding probabilities Na^+ and K^+ for the medium affinity state of the Trk system ($P_{Trk\_medium,Na}$ and $P_{Trk\_medium,K}$, respectively) are:

$$P_{Trk\_medium,Na}([K^+]_{ext},[Na^+]_{ext}) = \frac{[Na^+]_{ext} / Km_{Trk\_medium,Na}}{1+[K^+]_{ext} / Km_{Trk\_medium,K} + [Na^+]_{ext} / Km_{Trk\_medium,Na}}$$

(2.26) ,

$$P_{Trk\_medium,K}([K^+]_{ext},[Na^+]_{ext}) = \frac{[K^+]_{ext} / Km_{Trk\_medium,K}}{1+[K^+]_{ext} / Km_{Trk\_medium,K} + [Na^+]_{ext} / Km_{Trk\_medium,Na}}$$

(2.27) ,

where $Km_{Trk\_medium,K}$ and $Km_{Trk\_medium,Na}$ are the dissociation constants of the medium affinity state of the Trk system for K^+ and Na^+, respectively.

The characteristics of the Trk system in response to different physiological conditions

are not well understood. Therefore, we test several models that describe transporter activities based on how well the model agrees with published measurements for the Trk system. We firstly modeled the activity of the Trk system using the GHK equation. However, varying the activity of the Trk system in the resulting model did not alter the membrane potential in most conditions, which is contradictory to the report that activity of the Trk system has a significant impact on the membrane potential [41]. We then modeled the Trk system using the Nernst equation [35]. The behavior of the system is similar with that using the GHK equation, i.e. changes in Trk1,2p activity did not alter membrane potential. Finally, based on the evidence that the rate of K^+ influx does not depend on the extracellular K^+ concentration [7] and that the inward K^+ currents through the Trk system increases quadratically with the increase of the membrane potential [42], we model the K^+ inward flux through the Trk system to be proportional to the square of the membrane potential. Then, the following equations are used to describe the Na^+ and K^+ fluxes through the Trk system:

$$J_{Trk,Na} = (P_{Trk\_medium,Na} \cdot P_{Trk\_medium} + P_{Trk\_high,Na} \cdot P_{Trk\_high}) \cdot k_{Trk} \cdot Em^2,$$
$$J_{Trk,K} = (P_{Trk\_medium,K} \cdot P_{Trk\_medium} + P_{Trk\_high,K} \cdot P_{Trk\_high}) \cdot k_{Trk} \cdot Em^2$$

(2.28) ,

where k_{Trk} is the rate constant for the activity of Trk1,2p system.

2.2.6 NSC1 – non-selective cation channel

In addition to the Trk transport system, there exist other K^+ transporter systems, primarily a genetically uncharacterized non-selective cation channel, NSC1, that mediates low-affinity potassium uptake [43]. The contributions of these systems to cation homeostasis are less significant than that of the Trk system.

The probability of NSC1 binding to K^+ and Na^+ ($P_{NSC1,K}$ and $P_{NSC1,Na}$, respectively) are given by the CBP equations:

$$P_{NSC1,K}([K^+]_{ext},[Na^+]_{ext}) = \frac{[K^+]_{ext} / Km_{NSC1,K}}{1+[K^+]_{ext} / Km_{NSC1,K} + [Na^+]_{ext} / Km_{NSC1,Na}} \quad (2.29),$$

$$P_{NSC1,Na}([K^+]_{ext},[Na^+]_{ext}) = \frac{[Na^+]_{ext} / Km_{NSC1,Na}}{1+[K^+]_{ext} / Km_{NSC1,K} + [Na^+]_{ext} / Km_{NSC1,Na}} \quad (2.30),$$

where $Km_{NSC1,K}$ and $Km_{NSC1,Na}$ are dissociation constants.

Then, the K^+ and Na^+ fluxes through NSC1 were described following the equation describing a channel [35]:

$$J_{NSC1,K} = P_{NSC1,K} \cdot k_{NSC1} \cdot ([K^+]_{ext} - [K^+]_{cyt} \cdot \exp(\frac{F \cdot Em}{R \cdot T})) \quad (2.31),$$

$$J_{NSC1,Na} = P_{NSC1,Na} \cdot k_{NSC1} \cdot ([Na^+]_{ext} - [Na^+]_{cyt} \cdot \exp(\frac{F \cdot Em}{R \cdot T})) \quad (2.32),$$

where k_{NSC1} is a rate constant of NSC1.

2.3 H^+ production, import and export

2.3.1 H^+ production

Yeast cells generate a large number of H^+ ions during metabolism, which are pumped out of the cell by the H^+-ATPase, Pma1p. By creating a proton gradient across the plasma membrane, the proton flux builds up both the chemical energy (H+ gradient) and the electrical energy (membrane potential), which are important determinants for uptake of cations, amino acids, phosphates and other impermeable nutrient molecules [1,44].

In this study, to keep the model simple, we assume that the H^+ production rate (J_{H_prod}) is constant:

$$J_{H\_prod} = K_{H\_prod} \qquad (2.33)\ ,$$

where K_{H_prod} is a constant for H^+ production.

2.3.2 H^+ extrusion/Pma1p

H^+ is extruded into the external medium through the activity of Pma1p, an H^+-ATPase

localized on the yeast plasma membrane [45]. It plays an important role in nutrient uptake, regulation of membrane potential and pH homeostasis [46,47].

The chemical reaction for H^+ extrusion through Pma1p is:

$$[H^+]_{cyt} + [ATP] + [Pma1] \leftrightarrow [H^+]_{ext} + [ADP] + [Pi] + [Pma1],$$

where *[ATP]*, *[ADP]* and *[Pi]* are the concentrations of intracellular ATP, ADP and phosphate, respectively.

The H^+ flux across the plasma membrane (J_{Pma1}) is then can be written down as:

$$J_{Pma1} = k_+ \cdot [H^+]_{cyt} \cdot [ATP] - k_- \cdot [H^+]_{ext} \cdot [ADP] \cdot [Pi] \quad (2.34),$$

where k_+ and k_- are the rate constants for the forward and the backward chemical reactions shown above. Note that we assume that the amount of Pma1p in a cell is in excess.

The flux is driven by the energy difference between two sides of the equation, i.e. the Gibbs free energy. This difference (G_Δ) can be calculated as [35]:

$$G_\Delta = G_{0\_ATP} + R \cdot T \cdot \ln \frac{[H^+]_{ext} \cdot [ADP] \cdot [Pi]}{[H^+]_{\text{int}} \cdot [ATP]} - F \cdot Em \quad (2.35),$$

where G_{0_ATP} is the free energy released by ATP hydrolysis, *R* is the gas constant, *T* is the absolute temperature, *F* is the Faraday constant and *Em* is the membrane potential.

At equilibrium, both J_{Pma1} and G_{Δ} are equal to 0. By setting Eqn. (2.34) and (2.35) to 0 and solving these two equations, we find the ratio of the forward and backward constants (k_-/k_+) is related to the energy as follows:

$$\frac{k_-}{k_+} = \exp\left(\frac{G_{0_{ATP}} - F \cdot Em}{R \cdot T}\right) \quad (2.36).$$

Substituting Eqn. (2.36) into Eqn. (2.34), we get the flux through Pma1p for any given cytosolic and external H^+ concentrations:

$$J_{Pma1} = k_{Pma1} \cdot ([H^+]_{cyt} \cdot [ATP] - [H^+]_{ext} \cdot [ADP] \cdot [Pi] \cdot \exp(\frac{G_{0\_ATP} - F \cdot Em}{R \cdot T})) \quad (2.37).$$

Notice that here k_{Pma1} is equal to k_+, and its value is estimated from the experimentally measured Pma1p activity in ref. [45].

2.3.3 H^+ import

Nutrient molecules are transported into yeast cells through secondary transporters, and a majority of secondary transporters use the electrical and chemical potential created by the proton gradient across the plasma membrane as energy source for transportation [1,44,48]. Here, we assume the rate of H^+ uptake through secondary transporter activities is proportionate to the chemical and electrical energy. The chemical and

electrical energy created by the proton gradient is given by:

$$E_{EC} = R \cdot T \cdot \ln(\frac{[H^+]_{ext}}{[H^+]_{\text{int}}}) - Em \cdot F$$

Then, the rate of H^+ uptake can be approximated by:

$$J_{H\_uptake} = k_{H\_uptake} \cdot (R \cdot T \cdot \ln(\frac{[H^+]_{ext}}{[H^+]_{\text{int}}}) - Em \cdot F) \qquad (2.38),$$

where k_{H_uptake} is a rate constant.

2.4 Membrane leakage

In addition to the transport activity facilitated by different transporters, monovalent ions, such as H^+, K^+ and Na^+ are able to diffuse passively into and out of plasma membrane depending on the ion gradient and the membrane potential. In this study, we do not consider H^+ diffusion across the plasma membrane, since the rate of H^+ diffusion across plasma membrane is much lower than K^+ and Na^+, and thus, it can be ignored.

The passive diffusion of K^+ and Na^+ ions is given as follows

$$J_{diff,K/Na} = P_{K/Na} \cdot S_{Mem} \cdot ([K^+ / Na^+]_{ext} - [K^+ / Na^+]_{cyt} \cdot \exp(\frac{F \cdot Em}{R \cdot T})) \qquad (2.39),$$

where $P_{K/Na}$ is the permeability of the plasma membrane to K^+ or Na^+, respectively, and S_{Mem} is the surface area of the plasma membrane.

2.5 ODEs for ion transport

‘In the ODEs for ion transport, we track the changes in the total amount of each cation species, because the fluxes of the transporters modeled above are directly related to the changes in the amount of ions rather than the concentrations of ions in a cell. The concentration of each cation at a given time (*t*) can be calculated by dividing the amount of this cation by the volume of the osmotically changeable compartment at *t*.

The ODEs for the intracellular cations are:

$$
\begin{aligned}
\frac{d\,\mathrm{H}^+}{dt} &= -J_{Pma1} + \frac{3}{2}\cdot J_{Nha1,Na} + \frac{3}{2}\cdot J_{Nha1,K} + J_{H\_uptake} + J_{H\_production} \\
\frac{d\,Na^+}{dt} &= -J_{Ena1,Na} - J_{Nha1,Na} + J_{Trk,Na} + J_{NSC1,Na} + J_{diff,Na} \qquad (2.40)\ , \\
\frac{d\,K^+}{dt} &= -J_{Ena1,K} - J_{Nha1,K} + J_{Trk1,K} + J_{NSC1,K} - J_{Tok1} + J_{diff,K}
\end{aligned}
$$

where the coefficient 3/2 for the flux rate of Nha1p ($J_{Nha1,Na}$, $J_{Nha1,K}$) is added due to the fact that Nha1p is an electrogenic transporter and the assumption we made that 3 H^+ ions are taken in for every 2 K^+/Na^+ ions extruded by Nha1p.

3 Modeling transcriptional regulation

In order to understand the long-term adaptation processes to ionic stress conditions and, in particular, the significance of the induction of Ena1p in ionic stress responses, we model the activation of the HOG pathway, the calcineurin pathway and their regulation of *ENA1* expression in this section. It has been shown that that activation of the Snf1 pathway and the Rim101 pathway leads to Ena1p induction, partly through their inhibiton of the *ENA1* inhibitor, *NRG1* gene under alkaline pH condition [49]. Here we do not model these two pathways explicitly, since they are not well characterized quantitatively. Instead we model the inhibition of Nrg1p on ENA1 gene expression by assuming that it is dependent on external pH. Since we are primarily interested in the impact of ENA1 upregulation on the cellular physiological parameters rather than the detailed activation mechanism of ENA1 gene, this assumption is reasonable for the purpose of our study.

In this section, the ODE systems for each pathway and *ENA1* mRNA induction are described. The parameter values for each sub-module are either taken from existing models or chosen such that the simulation results are consistent with previously published data. The parameter values and references from which the parameters were obtained are listed in Table S3.

3.1 The HOG pathway

Under hyper-osmotic stress, activation of the HOG pathway leads to phosphorylation of the stress activated protein kinase Hog1p. Hog1p subsequently regulates glycerol production and export both at the post-translational level and the gene expression level to balance the external and internal osmolarity [20,21].

In this study, we adopt a previously published Hog1p model [50]. The model tracks the levels of phosphorylated and unphosphorylated forms of two members of the HOG pathway, Pbs2p, and Hog1p, the nuclear localization of Hog1p, the level of intracellular glycerol and the changes in the cell volume. The result of the model shows a good agreement with experimental observations. The ODEs from the model are shown below.

$$\frac{d[Pbs2]}{dt} = -\frac{K_{pho}^{Pbs2}\cdot[Pbs2]}{1+\left(\frac{P_{tur}}{\alpha_{Hog1}}\right)^{8}} + K_{depho}^{Pbs2}\cdot[Pbs2PP] - [Pbs2]\cdot V_{ratio}$$

$$\frac{d[Pbs2PP]}{dt} = \frac{K_{pho}^{Pbs2}\cdot[Pbs2]}{1+\left(\frac{P_{tur}}{\alpha_{Hog1}}\right)^{8}} - K_{depho}^{Pbs2}\cdot[Pbs2PP] - [Pbs2PP]\cdot V_{ratio}$$

$$\frac{d[Hog1c]}{dt} = -K_{pho}^{Hog1}\cdot[Pbs2PP]\cdot[Hog1c] + K_{depho}^{Hog1PPc}\cdot[Hog1PPc] - K_{imp}^{Hog1c}\cdot[Hog1c] + \frac{K_{exp}^{Hog1n}\cdot[Hog1n]\cdot V_{nuc}}{V_{cyt}} - [Hog1c]\cdot V_{ratio}$$

$$\frac{d[Hog1PPc]}{dt} = K_{pho}^{Hog1} \cdot [Pbs2PP] \cdot [Hog1c] - K_{depho}^{Hog1PPc} \cdot [Hog1PPc] - K_{imp}^{Hog1PPc} \cdot [Hog1PPc] + \frac{K_{exp}^{Hog1PPn} \cdot [Hog1PPn] \cdot V_{nuc}}{V_{cyt}} - [Hog1PPc] \cdot V_{ratio}$$

$$\frac{d[Hog1n]}{dt} = \frac{K_{imp}^{Hog1c} \cdot [Hog1c] \cdot V_{cyt}}{V_{nuc}} - K_{exp}^{Hog1n} \cdot [Hog1n] + K_{depho}^{Hog1PPn} \cdot [Hog1PPn] - [Hog1n] \cdot V_{ratio}$$

$$\frac{d[Hog1PPn]}{dt} = \frac{K_{imp}^{Hog1PPc} \cdot [Hog1PPc] \cdot V_{cyt}}{V_{nuc}} - K_{exp}^{Hog1PPn} \cdot [Hog1PPn] - K_{depho}^{Hog1PPn} \cdot [Hog1PPn] - [Hog1PPn] \cdot V_{ratio}$$

$$\frac{d[Gly_{\text{int}}]}{dt} = K_{s0}^{Glyc} + \frac{K_{s1}^{Glyc} \cdot (totalHog1PP)^4}{\beta_{Hog1}^4 + (totalHog1PP)^4} + K_{s2}^{Glyc} \cdot [Yt] - \left(K_{exp0}^{Glyc} + \frac{K_{exp1}^{Glyc} \cdot (P_{tur})^{12}}{(\gamma_{Hog1})^{12} + (P_{tur})^{12}} \right) \cdot [Gly_{\text{int}}] - [Gly_{\text{int}}] \cdot V_{ratio}$$

$$\frac{d[Yt]}{dt} = K_{s0}^{Yt} + K_{s1}^{Yt} \cdot [z4] - K_{t}^{Yt} \cdot [Yt] - [Yt] \cdot V_{ratio}$$

$$\frac{d[z1]}{dt} = \frac{4 \cdot ([Hog1PPn] - [z1])}{\tau}$$

$$\frac{d[z2]}{dt} = \frac{4 \cdot ([z1] - [z2])}{\tau}$$

$$\frac{d[z3]}{dt} = \frac{4 \cdot ([z2] - [z3])}{\tau}$$

$$\frac{d[z4]}{dt} = \frac{4 \cdot ([z3] - [z4])}{\tau}$$

$$V_{ratio} = \frac{dVolume_{cyt}}{Volume_{cyt} \cdot dt} = \frac{-G_{EK} \cdot Lp \cdot D_{\Pr essure}}{Volume_{cyt}}$$

$$totalHog1PP = \frac{([Hog1PPc(t)] \cdot V_{cyt} + [Hog1PPn(t)] \cdot V_{nuc}) \cdot 602}{6780};$$

(3.1).

The description of state variables in the Hog1 model is shown as following, and a detailed description of the model can be found in Zi et. al [50]:

Pbs2	unphosphorylated Pbs2p
Pbs2PP	phosphorylated Pbs2p
Hog1c	unphosphorylated cytoplasmic Hog1p
Hog1PPc	phosphorylated cytoplasmic Hog1p
Hog1n	unphosphorylated nuclear Hog1p
Hog1PPn	phosphorylated nuclear Hog1p
$Glyc_{int}$	intracellular glycerol
Yt	overall variable for the effect of transcriptional feedback intermediates on glycerol production
Vos	osmotic volume of the cell
z1, z2, z3, z4	linear chain variables for the delay of transcriptional feedback on glycerol production

For the parameters in the model, P_{tur} is the turgor pressure of the plasma membrane, which is defined later in Section 4; $Volume_{cyt}$ is the volume of the osmotically changeable compartment. Note that the impact of changes in cellular volume on protein concentrations is modeled using the term V_{ratio}, as in previous modeling studies [50,51],

G_{EK} is a geometrical factor relating the volume of a sphere to its surface, Lp is the hydraulic membrane permeability. $D_{Pressure}$ is the pressure difference between external pressure plus turgor pressure and the interior pressure of the cell plasma membrane, and its expression is given in Eqn (4.3) in Section 4. The rate of volume change of the osmotically changeable compartment is given in Section 4.

3.2 The calcineurin pathway

Under stress conditions, Ca^{2+} enters the cytosol from the external environment or the vacuole. Elevation of Ca^{2+} concentration activates the Ca^{2+} binding regulatory protein, calmodulin, and the Ca^{2+}-bound calmodulin subsequently binds to and activates calcineurin [52]. It has been shown that calcineurin activity is also under the negative regulation of Ppz proteins. The transcriptional response of the calcineurin pathway is mainly mediated by Crz1p. Upon activation, calcineurin dephosphorylates Crz1p, resulting in its nuclear localization. Crz1p subsequently regulates Ca^{2+} responsive gene targets, including *ENA1* [53].

3.2.1 Modeling intracellular Ca^{2+}

Intracellular Ca^{2+} homeostasis involves several Ca^{2+} specific transporters located at the plasma and the vacuolar membranes, as well as other feedback mechanisms, including regulation of calcineurin [54]. Since Ca^{2+} homeostasis is itself a highly complex process, a detailed model is beyond the scope of this study. We instead modeled the dynamics of intracellular Ca^{2+} concentration directly using an ordinary differential equation, based on the experimental data published in [24,25].

In general, the accumulation of cytosolic Ca^{2+} shows similar patterns under osmotic, saline and alkaline stress conditions. Under osmotic stress and saline stress, the cytosolic Ca^{2+} spikes in the first few minutes upon stress, and then, decreases rapidly to low levels [24,25]. The amplitude of this spike is determined by the extent of the stress. The level of cytosolic Ca^{2+} returns to the unstressed level in the long-term under osmotic stress conditions, while, under NaCl stress conditions, cytosolic Ca^{2+} reaches a steady state that is higher than the unstressed level. In addition, it has been shown that calcineurin mediates the transition of the Trk1,2p system from the medium affinity state to the high affinity state when intracellular Na^+ is excessive [55], which indirectly suggests that high level of intracellular Na^+ causes accumulation of cytosolic Ca^+. Therefore, in the model,

we assume that the long-term Ca^{2+} accumulation is dependent on both intracellular and extracellular Na^+ concentration. Under alkaline stress condition, cytosolic Ca^{2+} shows similar pattern to that seen under osmotic saline stresses. It spikes in the first few minutes and then returns to a lower level [24,25]. It has been shown that the calcineurin pathway is activated during alkaline stress, which in turn up-regulates *ENA1* expression [56]. Therefore, in the model, we assume that long-term intracellular Ca^{2+} concentration is also affected by the external pH, to model the impact of activation of the calcineurin pathway on *ENA1* expression.

Firstly, we model the initial Ca^{2+} spike by changing the initial cytosolic Ca^{2+} concentration at the beginning of the stress (t=0), as shown below:

$$\begin{aligned} &[Ca^{2+}]_{t=0} = K_{Ca,Na} + K_{Ca,pH} \\ &K_{Ca,Na} = \max((-2.5 + 0.002 \cdot Osmo_{Ext}), 0) \cdot 10^{-3} \qquad (3.2)\ , \\ &K_{Ca,pH} = \max((-2.0138 + 2.0138 \cdot pH_{Ext}), 0) \cdot 10^{-4} \end{aligned}$$

and $[Ca^{2+}]_{t=0}$ is the Ca^{2+} concentration at the start of stress, $Osmo_{Ext}$ is the external osmolarity and pH_{Ext} is the external pH. The parameter values in the expression are calculated by fitting the amplitude of the Ca^{2+} spike to experimental measurements in [24,25].

Secondly, the change in intracellular Ca^{2+} concentration is modeled as:

$$\frac{d\ [Ca^{2+}]}{dt} = C_{Ca} - d_{Ca} \cdot [Ca^{2+}] + k_{Ca,cyt} \cdot \frac{[Na^{+}]_{Cyt}^{h\_Na\_cyt}}{[Na^{+}]_{Cyt}^{h\_Na\_cyt} + Km_{Ca,cyt}^{h\_Na\_cyt}}$$
$$+ k_{Ca,ext} \cdot \frac{[Na^{+}]_{ext}^{h\_Na\_ext}}{[Na^{+}]_{ext}^{h\_Na\_ext} + Km_{Ca,ext}^{h\_Na\_ext}} + k_{Ca,pH} \cdot (pH_{ext} - 6.5) \qquad (3.3)\ ,$$
$$- 3 \cdot k_{CN,a} \cdot [\mathrm{Ca}^{2+}]^{3} \cdot [CN_{off}] + 3 \cdot k_{CN,da} \cdot [CN] - [Ca^{2+}] \cdot V_{ratio}$$

where C_{ca}, d_{Ca} are the Ca^{2+} production and degradation constants, respectively, $k_{Ca,cyt}$, $k_{Ca,ext}$ are the rate constants for the intra- and extra- cellular Na^{+} regulation on Ca^{2+} levels respectively, *h_Na_cyt, h_Na_ext* are the Hill-coefficients. $[CN_{off}]$ and *[CN]* are inactivated and activated calcineurin, respectively, which are described in the next section. V_{ratio} is given in Section 3.1 above.

The first two terms of the ODE above maintain the basal Ca^{2+} concentration in cells grown in unstressed conditions. The 3rd to 5th terms describes the long-term Ca^{2+} accumulation in response to high intra- and extra- cellular Na^{+}, external alkaline pH, respectively. The model results are consistent with previous experimental measurements on intracellular Ca^{2+} level during both salt stress and alkaline pH stress [24,25], and therefore, it is a good approximation to the adaptation processes investigated in this study.

3.2.2 Calcineurin activation

In the sub-model for the calcineurin pathway/calcineurin activation, we consider the

activation and deactivation of calcineurin by Ca^{2+} and Ppz proteins, respectively, as well as the calcineruin transcriptional regulation on Crz1p. The activation of a calcineurin molecule requires binding of three Ca^{2+} ions to a calmodulin molecule and subsequently to one calcineurin molecule. In the model, we simplify this two-step binding process by considering only the binding between Ca^{2+} and calcineurin, i.e. ignoring calmodulin. Since the focus of this study is to investigate the impact of *ENA1* gene induction through calcineurin, this simplification does not alter the main results in this study. Extending the model to incorporate calmodulin only alter the transient dynamics of the activated calcineurin slightly, and this does not change the conclusions of our study.

The system of ODEs is as follows:

$$\begin{aligned}
\frac{d\ [\mathrm{CN_{off}}]}{dt} &= -k_{CN,a}\cdot[\mathrm{Ca}^{2+}]^3\cdot[CN_{off}]+k_{CN,da}\cdot[CN]+k_{CN_Ppz,da}\cdot[Ppz]\cdot[CN]-[\mathrm{CN_{off}}]\cdot V_{ratio}\\
\frac{d\ [\mathrm{CN}]}{dt} &= k_{CN,a}\cdot[\mathrm{Ca}^{2+}]^3\cdot[CN_{off}]-k_{CN,da}\cdot[CN]-k_{CN_Ppz,da}\cdot[Ppz]\cdot[CN]-[\mathrm{CN}]\cdot V_{ratio}\\
\frac{d\ [\mathrm{Crz1}]}{dt} &= C_{Crz1}-d_{Crz1}\cdot[\mathrm{Crz1}]+k_{Crz1}\cdot[CN]-[\mathrm{Crz1}]\cdot V_{ratio}
\end{aligned} \quad (3.4).$$

Description of state variables and parameters:

[CN_{off}], [CN]	Concentrations of inactivated and activated calcineurin, respectively
[Crz1]	Concentration of Crz1p
$k_{CN,a}$, $k_{CN,da}$	Activation and deactivation rates of calcineurin, respectively
$k_{CNPpz,da}$	Deactivation rate of calcineurin by the Ppz phosphatase

C_{Crz1}, d_{Crz1}	The production rate and degradation rate of Crz1p, respectively
k_{Crz1}	The rate constant for calcineurin regulation on Crz1p.

3.3 Transcription regulation of Nrg1p

The transcriptional response to external alkaline environments is partly mediated by the repression of *NRG1* through several signaling pathways such as the Rim pathway and the Snf pathway [56]. Since *NRG1* encodes a transcription factor that inhibits *ENA1* expression, the increase in Ena1p expression under alkaline pH stress is partly due to the repression of *NRG1*. To examine the impact of *NRG1* repression on long-term ion homeostasis while making the model simple, (as mentioned above) we made the simplifying assumption that Nrg1p production decreases with increase in external pH. In this way, the long-term impact of Nrg1p on cellular ion regulation can be well approximated.

The ODE for Nrg1p is:

$$\frac{d\ [N\mathrm{rg1}]}{dt} = C_{Nrg1} \cdot \frac{Km_{Nrg1,pH}}{Km_{Nrg1,pH} + k_{pHext}} - d_{Nrg1} \cdot [N\mathrm{rg1}] - [Nrg1] \cdot V_{ratio} \qquad (3.5)\ ,$$

where C_{Nrg1} is the production rate constants of Nrg1p; $Km_{Nrg1,pH}$ is a Michealis-Menten constant describes the dependence of the level of Nrg1p on external pH (pH_{Ext});

$k_{pHext}=max(pH_{Ext}-6,0)$, this term describes that Nrg1p is repressed once external pH exceeds 6.0; and d_{Nrg1} is the degradation rate constants for Nrg1p. V_{ratio} is given in Section 3.1 above.

3.4 *ENA1* gene expression

ENA1 gene expression is under sophisticated regulation by several stress response pathways, including the HOG pathway, the calcineurin pathway and Nrg1p as described above [49]. This highlights the importance of Ena1p in stress adaptation processes. The HOG pathway and the calcineurin pathway regulate *ENA1* expression via transcription factors Sko1p and Crz1p, respectively. The equations describing the concentration of Crz1p under stress conditions are given in sections above. To keep the model simple, we do not model Sko1p explicitly. Instead, we assume that the level of Sko1p is proportional to the level of activated Hog1p in the nucleus. Hence, the ODEs for *ENA1* mRNA and Ena1p are:

$$\begin{aligned}\frac{d\ [E\mathrm{NA1}_{\mathrm{mRNA}}]}{dt} &= C_{ENA1,Nrg1}\cdot\frac{Km_{ENA1,Nrg1}^{h\_ENA1Nrg1}}{Km_{ENA1,Nrg1}^{h\_ENA1Nrg1}+[Nrg1]^{h\_ENA1Nrg1}}\\ &\quad + k_{ENA1,Crz1}\cdot[Crz1]+k_{ENA1,Hog1}\cdot[z2]-d_{ENA1mRNA}\cdot[E\mathrm{NA1}_{\mathrm{mRNA}}] \qquad (3.6)\ ,\\ \frac{d\ [E\mathrm{na1}]}{dt} &= kt_{Ena1}\cdot[E\mathrm{NA1}_{\mathrm{mRNA}}]-d_{Ena1}\cdot[E\mathrm{na1}]-[Ena1]\cdot V_{ratio}\end{aligned}$$

where, as before, V_{ratio} is given in Section 3.1 above.

Description of parameters:

$C_{ENA1,Nrg1}$	The production constant of *ENA1* mRNA
$Km_{ENA1,Nrg1}$	The Michaelis-Menten constant for Nrg1p repression of the *ENA1* gene
h_ENA1Nrg1	The Hill coefficient for Nrg1p repression of the *ENA1* gene
$k_{ENA1,Crz1}$	The up-regulation rate constant for the calcineurin pathway
$k_{ENA1,Hog1}$	The up-regulation rate constant for the HOG pathway
$d_{ENA1mRNA}$	The degradation rates for *ENA1* mRNA
kt_{Ena1}	The translation rate of *ENA1* mRNA
d_{Ena1}	The degradation rates for Ena1p

4 Modeling volume change

The change in cell volume is modeled to be dependent on the osmotic pressure difference across the plasma membrane, as in [51]. The equation is given as following:

$$\frac{d\ \mathrm{Volume}_{\mathrm{cyt}}}{dt} = -G_{EK} \cdot Lp \cdot D_{\mathrm{Pr}essure} \qquad (4.1)\ ,$$

where G_{EK} is a geometrical factor relating the volume of a sphere to its surface, *Lp* is the hydraulic membrane permeability and $D_{Pressure}$ is the pressure difference between external pressure plus turgor pressure and internal osmotic pressure.

Given the initial cell volume, turgor pressure is calculated as follows [57]:

$$P_{tur} = \max(P_{tur}^{0} \cdot (1 - \frac{Volume_{cyt}^{0} - Volume_{cyt}}{Volume_{cyt}^{0} - r_{Vol} \cdot Volume_{cyt}^{0}}),0) \qquad (4.2)\,,$$

where P_{tur}^{0}, $Volume_{cyt}^{0}$ are the turgor pressure and the cytosolic volume under normal growth conditions, respectively, and r_{vol} is the ratio of the cell volume with no turgor pressure over the cell volume in normal growth conditions.

Then, $D_{Pressure}$ can be calculated as follows:

$$\begin{aligned} D_{Pressure} &= P_{ext} + P_{tur} - P_{\text{int}} \\ &= ([Osmo_{Ext}] - [Osmo_{Cyt}]) \cdot R \cdot T + P_{tur} \end{aligned} \qquad (4.3)\,,$$

where

$$\begin{aligned} [Osmo_{Ext}] &= Osmo_{ext}^{0} - ([Na^{+}]_{ext}^{0} + [K^{+}]_{ext}^{0}) + ([Na^{+}]_{ext} + [K^{+}]_{ext}) \\ [Osmo_{Cyt}] &= (Osmo_{cyt}^{0} - ([Na^{+}]_{cyt}^{0} + [K^{+}]_{cyt}^{0})) \cdot \frac{Volume_{cyt}^{0}}{Volume_{cyt}} + ([Na^{+}]_{cyt} + [K^{+}]_{cyt}) + [Glycerol] \end{aligned}$$

(4.4).

P_{ext} and P_{int} are the external osmo-pressure and intracellular/cytosolic osmo-pressure, respectively. $Osmo_{ext}^{0}$ and $Osmo_{cyt}^{0}$ are the initial total osmolarity constants of the external medium and intracellular compartment, respectively. $[Na^{+}]_{ext}^{0}$, $[Na^{+}]_{cyt}^{0}$ are initial concentrations of extra- and intra-cellular Na^{+}, respectively, and $[K^{+}]_{ext}^{0}$, $[K^{+}]_{cyt}^{0}$ are initial concentrations of extra- and intra-cellular K^{+}, respectively. The parameter values in this section can be found in Table S4.

5 Modeling mutants

Strains without calcineurin activity

The intracellular responses in cells without calcineurin activity were examined in several simulations in this study. We model both the effect of FK506 treatment and calcineurin knockout strains by setting the activity of calcineurin (*[CN]* in the ODEs) to 0 in this study.

Other mutant strains

The simulations performed for other mutant strains in each figure are described below.

Figure 2B: The concentration of the Ppz phosphatase (*[Ppz]* in the ODEs) and the parameter $k_{ENA1,CN}$ (see Section 3.4) were set to be constant 0 during the simulation for the *ppz1,2* and the *crz1* mutant strains, respectively. Both the Ppz phosphatase concentration and $k_{ENA1,CN}$ were set to be constant 0 during the simulation for the *ppz1,2crz1* mutant strain.

Figure 3A: For each mutant strain, the activity of the deleted transporter (J_T in the ODEs, where *T* denote the transporter) is set to be 0 during the simulation.

Figure 3C: The concentration of free Ppz phosphatase (*[Ppz]* in the ODEs) is set to be equal to the total concentration of total amount of Ppz (Ppz^0) during the simulation for the *hal3* mutant strain (no inhibitory activity on Ppz phosphatase). The parameter Km_{Ppz}

(refer to Section 2.2.5) is set to be constant 0 during the simulation for the *HAL3* overexpression mutant, i.e. no Ppz phosphatase activity.

Figure 4A,C: The dependence of Tok1p activity on cytosolic phosphorylated Hog1p (P-Hog1p) is modeled as $\frac{Km_{Tok1,Hog1}^{3}}{[Hog1PPc]^{3}+Km_{Tok1,Hog1}^{3}}$ for inhibition, $\frac{[Hog1PPc]^{3}+Km_{Tok1,Hog1}^{3}}{Km_{Tok1,Hog1}^{3}}$ for activation and *1* for no interaction.

Figure 4B,D: For *nha1* and *tok1* mutant strains, Nha1p, Tok1p activities ($J_{Nha1,K}$, $J_{Nha1,Na}$ and J_{Tok1} in the ODEs) are set to be constant 0, respectively. For *hog1* mutant strain, the concentrations of all Hog1p moleculues are set to 0. For the hypothetical mutant strain (*mut1*), The activity of phosphorylated Hog1p at the cytosol (*Hog1PPc*) is set to 0.

Supplementary references

1. van der Rest ME, Kamminga AH, Nakano A, Anraku Y, Poolman B, et al. (1995) The plasma membrane of Saccharomyces cerevisiae: structure, function, and biogenesis. Microbiol Rev 59: 304-322.
2. Rodriguez-Navarro A (2000) Potassium transport in fungi and plants. Biochim Biophys Acta 1469: 1-30.
3. Goffeau A, Slayman CW (1981) The proton-translocating ATPase of the fungal plasma membrane. Biochim Biophys Acta 639: 197-223.
4. Ko CH, Gaber RF (1991) TRK1 and TRK2 encode structurally related K+ transporters in Saccharomyces cerevisiae. Mol Cell Biol 11: 4266-4273.
5. Gaber RF, Styles CA, Fink GR (1988) TRK1 encodes a plasma membrane protein required for high-affinity potassium transport in Saccharomyces cerevisiae. Mol Cell Biol 8: 2848-2859.
6. Rodriguez-Navarro A, Ramos J (1984) Dual system for potassium transport in Saccharomyces cerevisiae. J Bacteriol 159: 940-945.
7. Ramos J, Haro R, Rodriguez-Navarro A (1990) Regulation of potassium fluxes in Saccharomyces cerevisiae. Biochim Biophys Acta 1029: 211-217.
8. Ramos J, Rodriguez-Navarro A (1986) Regulation and interconversion of the potassium transport systems of Saccharomyces cerevisiae as revealed by rubidium transport. Eur J Biochem 154: 307-311.
9. Ruiz A, del Carmen Ruiz M, Sanchez-Garrido MA, Arino J, Ramos J (2004) The Ppz protein phosphatases regulate Trk-independent potassium influx in yeast. FEBS Lett 578: 58-62.
10. Lesage F, Guillemare E, Fink M, Duprat F, Lazdunski M, et al. (1996) A pH-sensitive yeast outward rectifier K+ channel with two pore domains and novel gating properties. J Biol Chem 271: 4183-4187.
11. Ketchum KA, Joiner WJ, Sellers AJ, Kaczmarek LK, Goldstein SA (1995) A new family of outwardly rectifying potassium channel proteins with two pore domains in tandem. Nature 376: 690-695.
12. Banuelos MA, Sychrova H, Bleykasten-Grosshans C, Souciet JL, Potier S (1998) The Nha1 antiporter of Saccharomyces cerevisiae mediates sodium and potassium efflux. Microbiology 144: 2749-2758.
13. Prior C, Potier S, Souciet JL, Sychrova H (1996) Characterization of the NHA1 gene encoding a Na+/H+-antiporter of the yeast Saccharomyces cerevisiae. FEBS Lett 387: 89-93.
14. Garciadeblas B, Rubio F, Quintero FJ, Banuelos MA, Haro R, et al. (1993)

Differential expression of two genes encoding isoforms of the ATPase involved in sodium efflux in Saccharomyces cerevisiae. Mol Gen Genet 236: 363-368.
15. Haro R, Garciadeblas B, Rodriguez-Navarro A (1991) A novel P-type ATPase from yeast involved in sodium transport. FEBS Lett 291: 189-191.
16. Hohmann S (2002) Osmotic stress signaling and osmoadaptation in yeasts. Microbiol Mol Biol Rev 66: 300-372.
17. Cyert MS (2003) Calcineurin signaling in Saccharomyces cerevisiae: how yeast go crazy in response to stress. Biochem Biophys Res Commun 311: 1143-1150.
18. Yenush L, Mulet JM, Arino J, Serrano R (2002) The Ppz protein phosphatases are key regulators of K+ and pH homeostasis: implications for salt tolerance, cell wall integrity and cell cycle progression. EMBO J 21: 920-929.
19. Posas F, Camps M, Arino J (1995) The PPZ protein phosphatases are important determinants of salt tolerance in yeast cells. J Biol Chem 270: 13036-13041.
20. Muzzey D, Gomez-Uribe CA, Mettetal JT, van Oudenaarden A (2009) A systems-level analysis of perfect adaptation in yeast osmoregulation. Cell 138: 160-171.
21. Westfall PJ, Patterson JC, Chen RE, Thorner J (2008) Stress resistance and signal fidelity independent of nuclear MAPK function. Proc Natl Acad Sci U S A 105: 12212-12217.
22. Mettetal JT, Muzzey D, Gomez-Uribe C, van Oudenaarden A (2008) The frequency dependence of osmo-adaptation in Saccharomyces cerevisiae. Science 319: 482-484.
23. Proft M, Struhl K (2004) MAP kinase-mediated stress relief that precedes and regulates the timing of transcriptional induction. Cell 118: 351-361.
24. Viladevall L, Serrano R, Ruiz A, Domenech G, Giraldo J, et al. (2004) Characterization of the calcium-mediated response to alkaline stress in Saccharomyces cerevisiae. J Biol Chem 279: 43614-43624.
25. Matsumoto TK, Ellsmore AJ, Cessna SG, Low PS, Pardo JM, et al. (2002) An osmotically induced cytosolic Ca2+ transient activates calcineurin signaling to mediate ion homeostasis and salt tolerance of Saccharomyces cerevisiae. J Biol Chem 277: 33075-33080.
26. Serrano R, Ruiz A, Bernal D, Chambers JR, Arino J (2002) The transcriptional response to alkaline pH in Saccharomyces cerevisiae: evidence for calcium-mediated signalling. Mol Microbiol 46: 1319-1333.
27. Nakamura T, Liu Y, Hirata D, Namba H, Harada S, et al. (1993) Protein phosphatase type 2B (calcineurin)-mediated, FK506-sensitive regulation of intracellular ions in yeast is an important determinant for adaptation to high salt stress conditions. EMBO J 12: 4063-4071.
28. Clotet J, Gari E, Aldea M, Arino J (1999) The yeast ser/thr phosphatases sit4 and

ppz1 play opposite roles in regulation of the cell cycle. Mol Cell Biol 19: 2408-2415.
29. Clotet J, Posas F, de Nadal E, Arino J (1996) The NH2-terminal extension of protein phosphatase PPZ1 has an essential functional role. J Biol Chem 271: 26349-26355.
30. Posas F, Casamayor A, Arino J (1993) The PPZ protein phosphatases are involved in the maintenance of osmotic stability of yeast cells. FEBS Lett 318: 282-286.
31. Yenush L, Merchan S, Holmes J, Serrano R (2005) pH-Responsive, posttranslational regulation of the Trk1 potassium transporter by the type 1-related Ppz1 phosphatase. Mol Cell Biol 25: 8683-8692.
32. de Nadal E, Clotet J, Posas F, Serrano R, Gomez N, et al. (1998) The yeast halotolerance determinant Hal3p is an inhibitory subunit of the Ppz1p Ser/Thr protein phosphatase. Proc Natl Acad Sci U S A 95: 7357-7362.
33. Mendoza I, Rubio F, Rodriguez-Navarro A, Pardo JM (1994) The protein phosphatase calcineurin is essential for NaCl tolerance of Saccharomyces cerevisiae. J Biol Chem 269: 8792-8796.
34. Lamb TM, Xu W, Diamond A, Mitchell AP (2001) Alkaline response genes of Saccharomyces cerevisiae and their relationship to the RIM101 pathway. J Biol Chem 276: 1850-1856.
35. Keener JP, Sneyd J (2009) Mathematical Phsiology I:Cellular Physiology: Springer.
36. Ohgaki R, Nakamura N, Mitsui K, Kanazawa H (2005) Characterization of the ion transport activity of the budding yeast Na+/H+ antiporter, Nha1p, using isolated secretory vesicles. Biochim Biophys Acta 1712: 185-196.
37. Kinclova-Zimmermannova O, Sychrova H (2006) Functional study of the Nha1p C-terminus: involvement in cell response to changes in external osmolarity. Curr Genet 49: 229-236.
38. Loukin SH, Saimi Y (1999) K(+)-dependent composite gating of the yeast K(+) channel, Tok1. Biophys J 77: 3060-3070.
39. Haro R, Rodriguez-Navarro A (2002) Molecular analysis of the mechanism of potassium uptake through the TRK1 transporter of Saccharomyces cerevisiae. Biochim Biophys Acta 1564: 114-122.
40. Casado C, Yenush L, Melero C, Ruiz Mdel C, Serrano R, et al. (2010) Regulation of Trk-dependent potassium transport by the calcineurin pathway involves the Hal5 kinase. FEBS Lett 584: 2415-2420.
41. Madrid R, Gomez MJ, Ramos J, Rodriguez-Navarro A (1998) Ectopic potassium uptake in trk1 trk2 mutants of Saccharomyces cerevisiae correlates with a highly hyperpolarized membrane potential. J Biol Chem 273: 14838-14844.
42. Kuroda T, Bihler H, Bashi E, Slayman CL, Rivetta A (2004) Chloride channel function in the yeast TRK-potassium transporters. J Membr Biol 198: 177-192.

43. Ko CH, Buckley AM, Gaber RF (1990) TRK2 is required for low affinity K+ transport in Saccharomyces cerevisiae. Genetics 125: 305-312.
44. Goossens A, de La Fuente N, Forment J, Serrano R, Portillo F (2000) Regulation of yeast H(+)-ATPase by protein kinases belonging to a family dedicated to activation of plasma membrane transporters. Mol Cell Biol 20: 7654-7661.
45. Serrano R (1983) In vivo glucose activation of the yeast plasma membrane ATPase. FEBS Lett 156: 11-14.
46. Perlin DS, Brown CL, Haber JE (1988) Membrane potential defect in hygromycin B-resistant pma1 mutants of Saccharomyces cerevisiae. J Biol Chem 263: 18118-18122.
47. Eraso P, Gancedo C (1987) Activation of yeast plasma membrane ATPase by acid pH during growth. FEBS Lett 224: 187-192.
48. Jennings DH (1995) The physiology of fungal nutrition Cambridge University Press. 640 p.
49. Ruiz A, Arino J (2007) Function and regulation of the Saccharomyces cerevisiae ENA sodium ATPase system. Eukaryot Cell 6: 2175-2183.
50. Zi Z, Liebermeister W, Klipp E (2010) A quantitative study of the Hog1 MAPK response to fluctuating osmotic stress in Saccharomyces cerevisiae. PLoS One 5: e9522.
51. Klipp E, Nordlander B, Kruger R, Gennemark P, Hohmann S (2005) Integrative model of the response of yeast to osmotic shock. Nat Biotechnol 23: 975-982.
52. Cyert MS (2001) Genetic analysis of calmodulin and its targets in Saccharomyces cerevisiae. Annu Rev Genet 35: 647-672.
53. Stathopoulos AM, Cyert MS (1997) Calcineurin acts through the CRZ1/TCN1-encoded transcription factor to regulate gene expression in yeast. Genes Dev 11: 3432-3444.
54. Cunningham KW, Fink GR (1994) Ca2+ transport in Saccharomyces cerevisiae. J Exp Biol 196: 157-166.
55. Haro R, Banuelos MA, Quintero FJ, Rubio F, Rodriguez-Navarro A (1993) Genetic basis of sodium exclusion and sodium tolerance in yeast. A model for plants. Physiologia Plantarum 89: 868-874.
56. Platara M, Ruiz A, Serrano R, Palomino A, Moreno F, et al. (2006) The transcriptional response of the yeast Na(+)-ATPase ENA1 gene to alkaline stress involves three main signaling pathways. J Biol Chem 281: 36632-36642.
57. Zimmermann U (1978) Physics of turgor- and osmoregulation. Ann Rev Plant Physiol 29: 121-148.
